# Enhanced Sequence-Activity Mapping and Evolution of Artificial Metalloenzymes by Active Learning

**DOI:** 10.1021/acscentsci.4c00258

**Published:** 2024-05-22

**Authors:** Tobias Vornholt, Mojmír Mutný, Gregor W. Schmidt, Christian Schellhaas, Ryo Tachibana, Sven Panke, Thomas R. Ward, Andreas Krause, Markus Jeschek

**Affiliations:** † Department of Biosystems Science and Engineering, ETH Zurich, Mattenstrasse 26, 4058 Basel, Switzerland; ‡ National Centre of Competence in Research (NCCR) Molecular Systems Engineering, 4056 Basel,Switzerland; § Department of Computer Science, ETH Zurich, Andreasstrasse 5, 8092 Zurich, Switzerland; ∥ Department of Chemistry, University of Basel, Mattenstrasse 24a, 4058 Basel, Switzerland; ⊥ Institute of Microbiology, University of Regensburg, Universitätsstraße 31, 93053 Regensburg, Germany

## Abstract

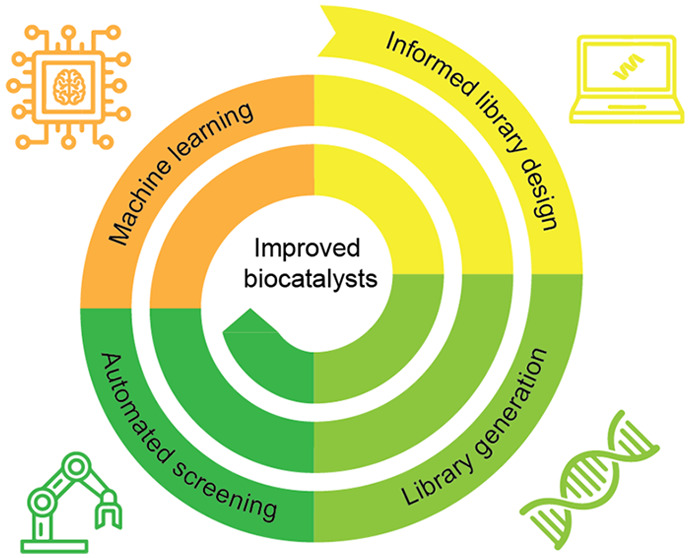

Tailored enzymes are crucial for the transition to a sustainable bioeconomy. However, enzyme engineering is laborious and failure-prone due to its reliance on serendipity. The efficiency and success rates of engineering campaigns may be improved by applying machine learning to map the sequence-activity landscape based on small experimental data sets. Yet, it often proves challenging to reliably model large sequence spaces while keeping the experimental effort tractable. To address this challenge, we present an integrated pipeline combining large-scale screening with active machine learning, which we applied to engineer an artificial metalloenzyme (ArM) catalyzing a new-to-nature hydroamination reaction. Combining lab automation and next-generation sequencing, we acquired sequence-activity data for several thousand ArM variants. We then used Gaussian process regression to model the activity landscape and guide further screening rounds. Critical characteristics of our pipeline include the cost-effective generation of information-rich data sets, the integration of an explorative round to improve the model’s performance, and the inclusion of experimental noise. Our approach led to an order-of-magnitude boost in the hit rate while making efficient use of experimental resources. Search strategies like this should find broad utility in enzyme engineering and accelerate the development of novel biocatalysts.

## Abstract 2

Large-scale, automated screening and active machine learning can increase the success rate and efficiency of enzyme engineering.

## Introduction

Biocatalysis and metabolic engineering offer sustainable production routes for many compounds of interest and thus hold the potential to transform various industries. However, extensive enzyme engineering is typically required to obtain a suitable biocatalyst for a desired application. This is often a time-consuming, empirical process whose outcome is subject to chance, as classical methods are agnostic to the topology of the underlying sequence-activity landscape. Engineering strategies that incorporate machine learning to model this landscape could render enzyme engineering more efficient and increase the likelihood of identifying an optimal solution. Accordingly, machine learning-assisted directed evolution (MLDE) has attracted significant attention in recent years.^1−3^

In general, MLDE starts with an initial screening round in which both sequence and activity are recorded for a number of enzyme variants. These sequence-activity data are then used to train a machine learning model, with the objective of predicting the activity of untested variants directly from their sequence. If successful, such models can suggest variants that are likely to be highly active and thus support further screening rounds by in silico library design.^1^ Further, the model can be iteratively updated with new data to improve its predictive performance, a strategy referred to as active learning. While several studies have demonstrated the general feasibility of such approaches,^4−12^ there are still various challenges that need to be addressed to maximize the success rate and efficiency of MLDE and enable its widespread implementation. This pertains to various aspects such as library design, experimental data acquisition, model development, and the strategy for sampling the sequence space.

With regard to library design, the crucial challenge is to create a library that is as information-dense as possible to allow for the development of accurate models while keeping the screening effort manageable. In the initial stages of model development, this calls for libraries that exhibit a high degree of sequence diversity to provide adequate information on the underlying sequence space, while at the same time containing a sufficient number of active mutants.^13^ These requirements can be difficult to reconcile, as simultaneous randomization of multiple residues commonly results in a large fraction of inactive mutants, from which little to no meaningful information for model training can be extracted.

Once a library has been generated, it is often challenging to measure a sufficiently large set of sequence-activity data. In some cases, high-throughput assays such as fluorescence-activated cell sorting can be combined with deep sequencing to obtain very large data sets.^14,15^ However, most enzymatic reactions of industrial relevance require more laborious analytical procedures to obtain a readout for activity. Moreover, the need to also obtain sequence information on all tested variants can lead to prohibitive costs if conventional Sanger sequencing is used. Consequently, most studies to date have relied on small data sets (10^1^–10^2^ variants).^4−10^ While this has led to several successful demonstrations of MLDE, larger data sets are likely to lead to more accurate machine learning models and improve the chances of identifying variants with the desired properties,^11^ particularly as the search space increases in size.

Beyond these experimental considerations, several critical decisions have to be made regarding the machine learning strategy. Prominent examples in this regard include the encoding strategy for the protein sequences and the choice of a suitable machine learning algorithm. Many encoding strategies have been suggested for creating a meaningful representation of protein variants, ranging from simple one-hot encoding and descriptors based on amino acid properties^16−18^ to structure-based descriptors^19,20^ and learned embeddings.^21,22^ Similarly, various machine learning algorithms have been employed or suggested for MLDE, including linear regression,^23−25^ Gaussian processes,^4,7−9,25,26^ and neural networks.^12^ While the best strategy depends on the data set and task at hand, Gaussian processes have repeatedly revealed their utility for active learning.^8,9,25^

Less attention has been devoted to other aspects of the machine learning process, such as the handling of experimental noise or the sampling strategy during ML-guided screening rounds, both of which are critical to the success and efficiency of MLDE. With regard to the sampling strategy, many studies have relied on a single training phase followed by greedy sampling of the top predictions of the resulting model. Due to inevitable biases in library generation and the limitations in generating sufficient sequence-activity data, this is unlikely to result in a comprehensive and accurate representation of the sequence-activity landscape. Consequently, such models may be “blind” for promising regions of the sequence space, leading to suboptimal outcomes such as low hit rates. Active learning strategies that improve the model in iterative cycles of experiments and machine learning may help to develop a better representation of the sequence-activity landscape, as these can converge to the optimal solution over time.^27^ However, the aforementioned bottleneck in experimental data generation makes performing many iterations undesirable. Thus, resources invested into model improvement (i.e., exploration) must be carefully weighed against the focus on regions of the sequence space that are likely to contain active variants but might only comprise local optima (exploitation). In addition, activity may not be the only selection criterion during exploitation. Instead, it is often desirable to sample various potential optima to obtain a diverse set of variants, which requires more elaborate approaches than simple greedy selection of top predictions.^28^ Hence, smart sampling strategies for active learning are required to maximize the chances of success at a given experimental budget.

In this study, we introduce an integrated experimental and computational pipeline that addresses critical limitations in the MLDE of enzymes. Specifically, we combine informed library design with large-scale screening and a novel active machine-learning strategy. As an impactful testbed, we selected an artificial metalloenzyme (ArM) for gold-catalyzed hydroamination, a new-to-nature reaction for atom-economical C–N bond formation. We simultaneously engineered five crucial amino acid residues in this ArM, corresponding to a search space of 3 200 000 possible variants. To sample this space, we combined lab automation with a cost-efficient next-generation sequencing (NGS) strategy, which allowed us to acquire sequence-activity data on more than 2000 ArM variants. Furthermore, we developed a machine learning model based on Gaussian process regression that incorporates optimized descriptors and estimates of experimental noise to efficiently navigate the sequence space. Guided by the model’s uncertainty estimates, we performed a second screening round focused on exploration and model refinement. Importantly, our results demonstrate that this targeted exploration substantially improved the model’s performance. The optimized model reliably proposed highly active ArM variants in a final exploitation round, as illustrated by a 12-fold increased hit rate compared to the initial library.

## Results

### Design of an Information-Dense ArM Library

ArMs are hybrid catalysts that promise to significantly increase the number of reactions available in biocatalysis by equipping enzymes with the catalytic versatility of abiological transition metal cofactors.^29^ ArMs have been created for a variety of natural and non-natural reactions,^30−35^ and some have demonstrated catalytic prowess comparable to that of natural enzymes.^36−39^ However, most ArMs initially display a low activity, and extensive protein engineering is required to identify catalytically proficient variants. This engineering is typically a labor-intensive and slow process. Therefore, ArMs represent an impactful yet challenging use case for MLDE.

A particularly versatile strategy for creating ArMs is to incorporate an organometallic cofactor into the tetrameric protein streptavidin (Sav) using a biotin moiety as the anchor. Using this approach, we have previously engineered an ArM for gold-catalyzed hydroamination by exhaustively screening a library of 400 Sav double mutants (Sav S112X K121X) using a whole-cell assay in 96-well plates.^40^ While this represents an attractive starting point, extending the search space to more positions offers the opportunity to achieve further improvements, which will be crucial for adapting ArMs for real-world applications. However, exhaustive screening quickly becomes intractable in this case, and smart heuristics for the efficient exploration of the underlying sequence-activity landscape are essential.^41^

**Figure 1:**
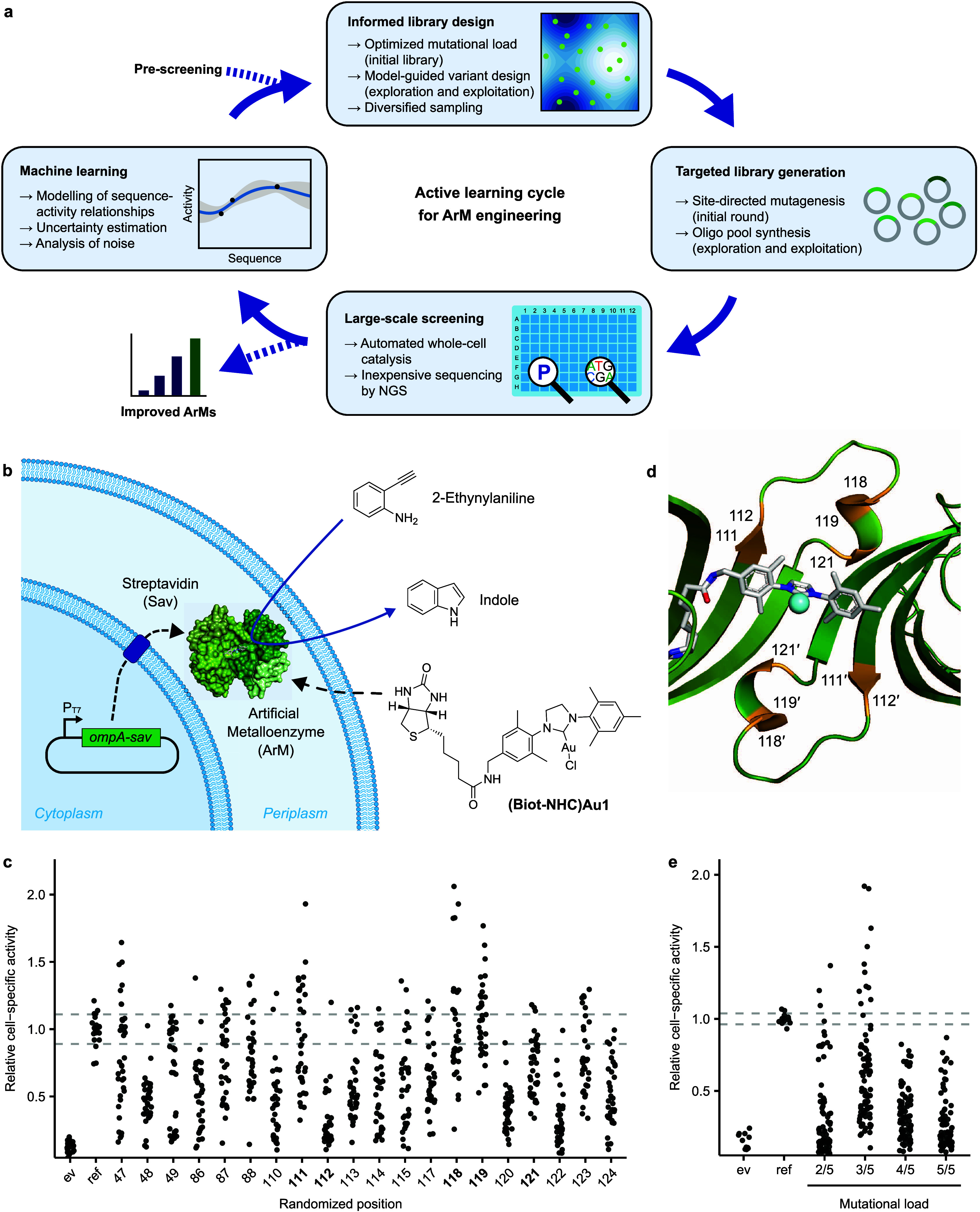
Engineering strategy and library design for ArMs catalyzing hydroamination. a. Illustration of the active learning strategy for ArM engineering. An iterative process of library design, cloning, large-scale screening, and machine learning was used to model the sequence-activity landscape and identify improved ArMs. Crucial steps and considerations are highlighted and are explained in the main text. b. Illustration of whole-cell biocatalysis using an ArM in the periplasm of *E. coli*. Sav is exported to the periplasm by means of an N-terminal OmpA signal peptide, where it binds the biotinylated cofactor **(Biot-NHC)Au1**. The resulting ArM converts 2-ethynylaniline to indole in a new-to-nature hydroamination reaction. Indole can subsequently be quantified using a colorimetric assay. c. Single site-saturation mutagenesis to identify influential amino acid residues with respect to ArM activity. Starting from the reference variant Sav S112F K121Q, 20 residues in Sav were individually mutated using degenerate NDT codons. The activity of the resulting variants is displayed relative to the mean activity of the reference variant (“ref”). Dashed lines indicate one standard deviation around the mean activity of the reference variant, which was measured in triplicate in each 96-well plate. A strain lacking Sav, i.e., containing an empty vector (“ev”), was included as a control (*n* = 3 per 96-well plate). The five positions selected for combinatorial randomization are highlighted in bold. Note that no improvement was expected at positions 112 and 121, as the reference variant had already been optimized with regard to these positions.^40^ d. Residues selected for randomization (highlighted in orange) in a ribbon model of Sav harboring a metathesis catalyst (PDB 5IRA). For clarity, only two biotin-binding sites of two opposing Sav monomers (a so-called functional dimer) are displayed. e. Effect of different multisite randomization strategies on the activity distribution of ArM libraries. Starting from the reference variant, either two, three, four or five residues among positions 111, 112, 118, 119, and 121 were randomized simultaneously. Hydroamination activity is displayed relative to the average activity of the reference variant (“ref”, *n* = 3 per 96-well plate) for 90 variants from each library. A strain containing an empty vector (“ev”) was included as a control (*n* = 3 per 96-well plate).

To navigate the sequence-activity landscape of the ArM, we devised an iterative active learning cycle involving library design, cloning, screening, and machine learning (Figure 1a). With regard to library design, the first step is to choose the target residues and a randomization scheme. To maximize the potential impact of the screening campaign, we aimed to find important positions in Sav besides the previously identified residues S112 and K121.^40^ Thus, we individually randomized the 20 residues closest to the biotinylated gold cofactor in Sav S112F K121Q, which is the most active variant we had observed before^40^ (referred to as “reference variant” herein). Randomization was performed using degenerate NDT (N = A, C, G or T; D = A, G or T) codons, which encode 12 amino acids covering all chemical classes of amino acids, a strategy that has revealed high success rates at a reduced screening effort.^40^ Subsequently, we measured hydroamination activity using our previously established protocol relying on periplasmic catalysis in *Escherichia coli* (Figure 1b).^40^ We tested 36 clones per randomized position to achieve a statistical library coverage of approximately 95%.^42^ As expected, most variants displayed reduced activity compared to the reference variant (Figure 1c). Notably, positions 111, 118, and 119 revealed the highest potential for improvement upon mutagenesis, with several variants outperforming the reference variant. Consequently, we selected these positions for further engineering. In addition, we chose to also randomize positions 112 and 121 again, as our observations had indicated that epistatic effects play an important role in highly active ArM mutants.^40^

Next, we sought to create a combinatorial library of the five selected positions (111, 112, 118, 119, and 121, Figure 1d), which, upon full randomization, corresponds to a search space of 20^5^ = 3 200 000 variants. This greatly exceeds the capacity of typical activity assays and well plate-based screenings. Thus, navigating the underlying sequence-activity landscape represents a significant challenge. In order to model this space for MLDE, it is crucial to design a library that offers a good coverage of the targeted sequence space and at the same time maintains a sufficient proportion of active variants.^13^ While simultaneous randomization of all five residues would fulfill the first criterion, we anticipated that the high mutational load would likely lead to a large fraction of inactive variants. This would not only diminish the chances of identifying improved variants but also, importantly, would be uninformative for machine learning. Upon initial tests, we indeed observed a marked drop in the activity distribution when randomizing more than three of the five positions simultaneously (Figure 1e). Accordingly, we set out to construct a library with three to four mutations distributed across the five target residues as a good compromise between high sequence-diversity and sufficient residual activity. In other words, the constructed library covers all five target positions, but individual variants contain at most four amino acid substitutions relative to the reference variant Sav S112F K121Q, which served as the parent of the library (Figure S1 of the Supporting Information). This was achieved by site-directed mutagenesis PCR using various sets of primers containing degenerate NNK (K = G or T) codons at different positions and subsequent mixing of the resulting sublibraries (see Methods).

### Large-Scale Acquisition of Sequence-Activity Data

Our previously established whole-cell screening protocol for ArMs relied on periplasmic Sav expression, ArM assembly, and catalysis in 96-well plate format. By combining this protocol with conventional Sanger sequencing, we were able to obtain sequence-activity data for a few hundred variants.^40^ Although this platform was more flexible and simpler than comparable screening strategies involving protein purification, it still required considerable manual labor, particularly for product quantification. Additionally, when larger data sets are required, Sanger sequencing rapidly leads to prohibitively high sequencing costs. To facilitate the generation of larger data sets for MLDE, we thus sought to minimize manual intervention in the activity assay and develop more cost-efficient means of obtaining the sequence information for each functionally characterized variant.

**Figure 2:**
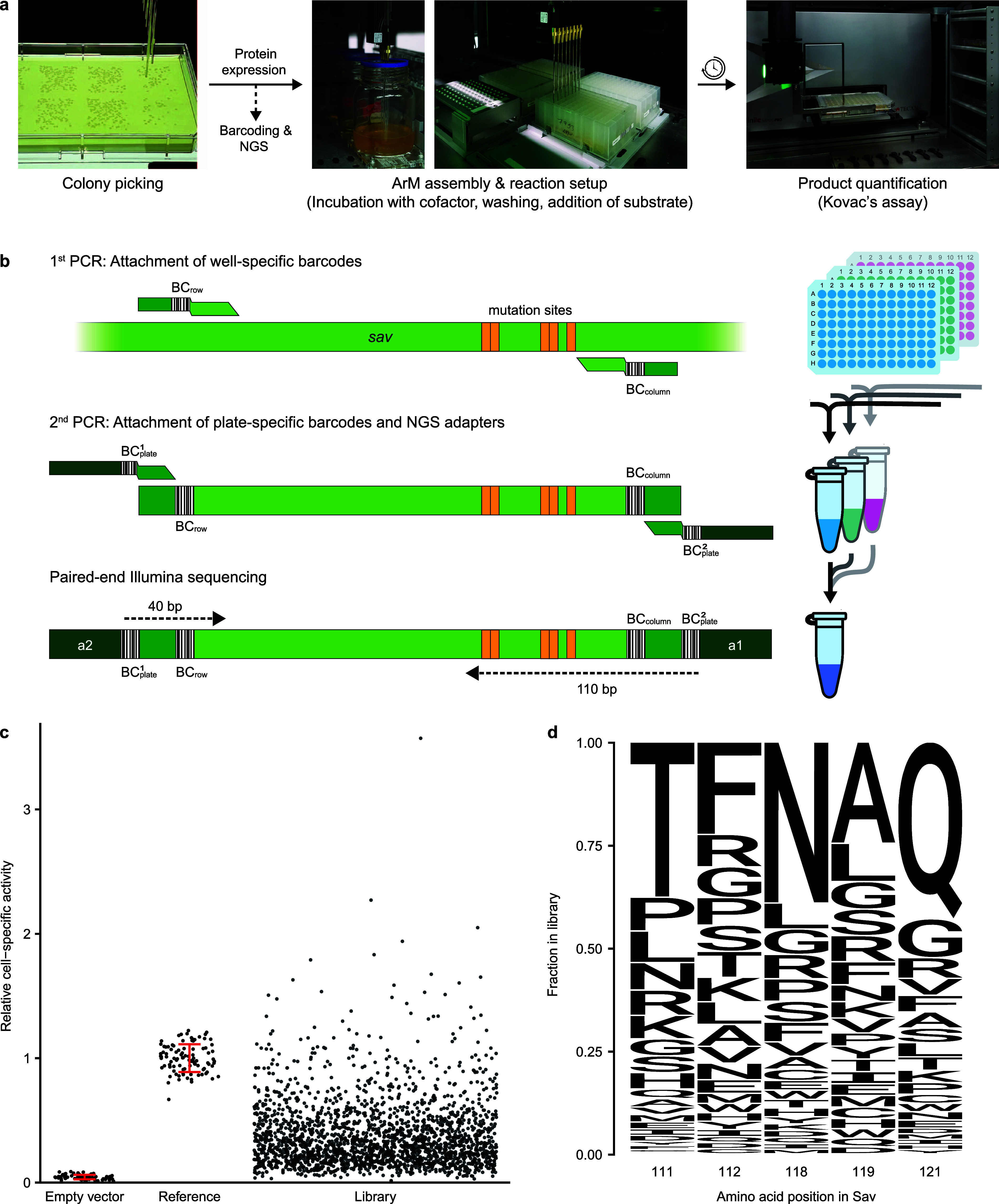
Large-scale acquisition of sequence-activity data for ArMs. a. Depiction of the critical automated steps in the screening workflow. Colony picking, ArM assembly, reaction setup, and product quantification were performed on a lab automation platform. The less labor-intensive protein expression protocol was performed manually. In parallel to the activity assay, samples of the starter cultures were processed further for NGS. b. PCR-based barcoding strategy for cost-effective sequencing of Sav variants in 96-well plates by NGS. First, the mutated region of the Sav gene is amplified using primers with row- (BC_row_) and column-specific (BC_column_) DNA barcodes. This step is performed in PCR plates using heat-treated bacterial cultures as templates. After pooling all samples from one plate, a second PCR is performed to add two plate-specific barcodes (BC_plate_) as well as adapters required for Illumina sequencing (a1 and a2). Subsequently, all samples are pooled and sequenced via paired-end reading to cover all barcodes and mutation sites. c. Cell-specific hydroamination activity of 2164 ArM variants from the initial library obtained by automated screening of 32 96-well plates. Only variants that were included for model training are displayed. Controls (empty vector and reference variant) are displayed with their standard deviation in red. d. Fraction of amino acids at the five randomized positions in Sav. Note that the amino acids of the reference variant (Sav 111T 112F 118N 119A 121Q, abbreviated Sav TFNAQ) are the most abundant, as the library was derived from this variant and contained at most four amino acid substitutions per variant.

First, we automated all steps in the assay protocol that are labor-intensive (and thus limiting in terms of throughput) or critical for reproducibility. Specifically, we made use of a Tecan EVO 200 platform for all steps from colony picking to product quantification, with the exception of Sav expression in 96-deep well plates, which only requires a small number of pipetting steps (Figure 2a). The most important addition to our previous semiautomated pipeline^40^ is the photometric quantification of the product indole. While this is a laborious procedure when carried out manually, the automated version simplifies screenings and proved to be very reproducible (Figure S2). As the robotic platform can handle up to eight 96-well plates at the same time, it greatly accelerates the acquisition of large data sets.

Besides the activity assay, another critical barrier to obtaining sufficiently large sets of sequence-activity data can be the cost of sequencing. Obtaining the sequences of several thousand protein variants by Sanger sequencing typically costs more than USD 10 000, which is prohibitive for most academic laboratories. In principle, the cost per variant can be reduced significantly by relying on NGS, which quickly becomes more cost-efficient than Sanger sequencing as the library size increases. However, in NGS all variants are sequenced in bulk, which means a method to retroactively link each sequence to the corresponding activity measurement is required. Previously, the use of DNA barcodes has been suggested to enable NGS of protein variants distributed across 96-well plates.^43−45^ Building on these strategies, we established a two-step PCR protocol for the barcoding of Sav variants that is compatible with the Illumina NGS platforms (Figure 2b). In the first step, which is carried out in 96-well plates, the randomized region of the Sav gene is amplified using primers that append a well-specific barcode combination as well as constant regions to the ends of the PCR products. This is achieved using eight forward (representing the plate‘s rows) and 12 reverse primers (representing the columns). For simplicity, heat-treated samples of bacterial cultures serve as templates, avoiding the need for laborious and costly plasmid purification.

Subsequently, PCR products are pooled by plate, and each pool is gel-purified and used as a template for a second PCR. In this step, primers binding to the previously added terminal constant regions are used for amplification. These primers contain overhangs to append plate-specific barcodes as well as the adapters required for NGS. Through the combination of well- (1^st^ step) and plate-specific (2^nd^ step) barcodes, it is possible to sequence thousands of variants from multiple plates in a single, low-cost NGS run and to assign the obtained sequences to the corresponding activity value obtained in the functional assay. In our specific case, paired-end sequencing of 40 bp from one end and 110 bp from the other end of the final PCR product was sufficient to read all well- and plate-specific barcodes as well as the five mutation sites in the Sav gene at a high read coverage (average of >100-fold per variant) and low cost (see Discussion).

Relying on the combination of automated activity assay and NGS, we screened 32 96-well plates containing variants from the aforementioned library of Sav. As each plate contained six controls (empty vector and reference variant in triplicate), this amounts to a total of 2880 variants. Excluding mutants that failed to grow, we obtained activity data on 2790 variants. Most of these displayed an intermediate activity between the background level of cells lacking Sav (empty vector) and the reference variant Sav S112F K121Q (Figure 2c). Notably, approximately 3% of all mutants were more active than the reference. Using the NGS-based strategy, we retrieved the sequences for 2663 out of 2880 wells containing Sav mutants. After excluding variants with nonsense mutations and wells containing more than one variant, sequence-activity data for 2164 clones were obtained, of which 2035 were distinct variants. Notably, for variants appearing in multiple wells, the deviation between these replicate activity measurements was generally low, corroborating the high robustness of the assay (Figure S3). Importantly, the library displayed a high degree of sequence diversity, with every amino acid appearing in every position (Figure 2d) and an average Hamming distance of 4.3 between the mutants. Note that the amino acids of the reference variant were the most abundant in each position, as we did not randomize all five positions simultaneously. Thus, the library exhibited a high degree of variability both in terms of activity distribution (including a low fraction of inactive variants) as well as sequence diversity. This indicated that the aforementioned design goals for the library were met, providing a promising data basis for modeling the sequence-activity landscape by machine learning.

As we had previously recorded sequence-activity data for 400 Sav double mutants (S112X K121X) that are part of the same sequence space,^40^ we added these older data to the measurements obtained herein. As a result, a total of 2992 data points covering 2435 distinct ArM variants were available as initial training data for machine learning.

### Development of an Initial Machine Learning Model of ArM Activity

To construct a model that can reliably predict the activity of untested ArM variants and guide further screening rounds, we relied on Gaussian process (GP) regression.^46^ This machine learning technique can capture highly nonlinear relationships and has the distinct advantage of being probabilistic, which means that it predicts a probability distribution rather than a point estimate, and thus provides an estimate for the confidence of each prediction. This feature can not only help users assess the uncertainty of individual predictions, but also is ideally suited for active learning strategies. In this scenario, the model’s uncertainty estimates can be used to guide subsequent screening rounds toward uncertain regions of sequence space with the goal of improving the model (i.e., exploration), before suggesting highly active variants in later rounds (i.e., exploitation).

GPs are characterized by a mean and a covariance function, which is commonly referred to as kernel. In our case, as we operate on the space of protein sequences, the kernel measures the similarity between different ArM variants. Since the selection of a suitable kernel is of paramount importance for good performance and sample efficiency (i.e., predicting accurately with little data), we performed a benchmarking process and found that the nonlinear Matérn kernel^46^ performed best in our case (see Methods).

**Figure 3:**
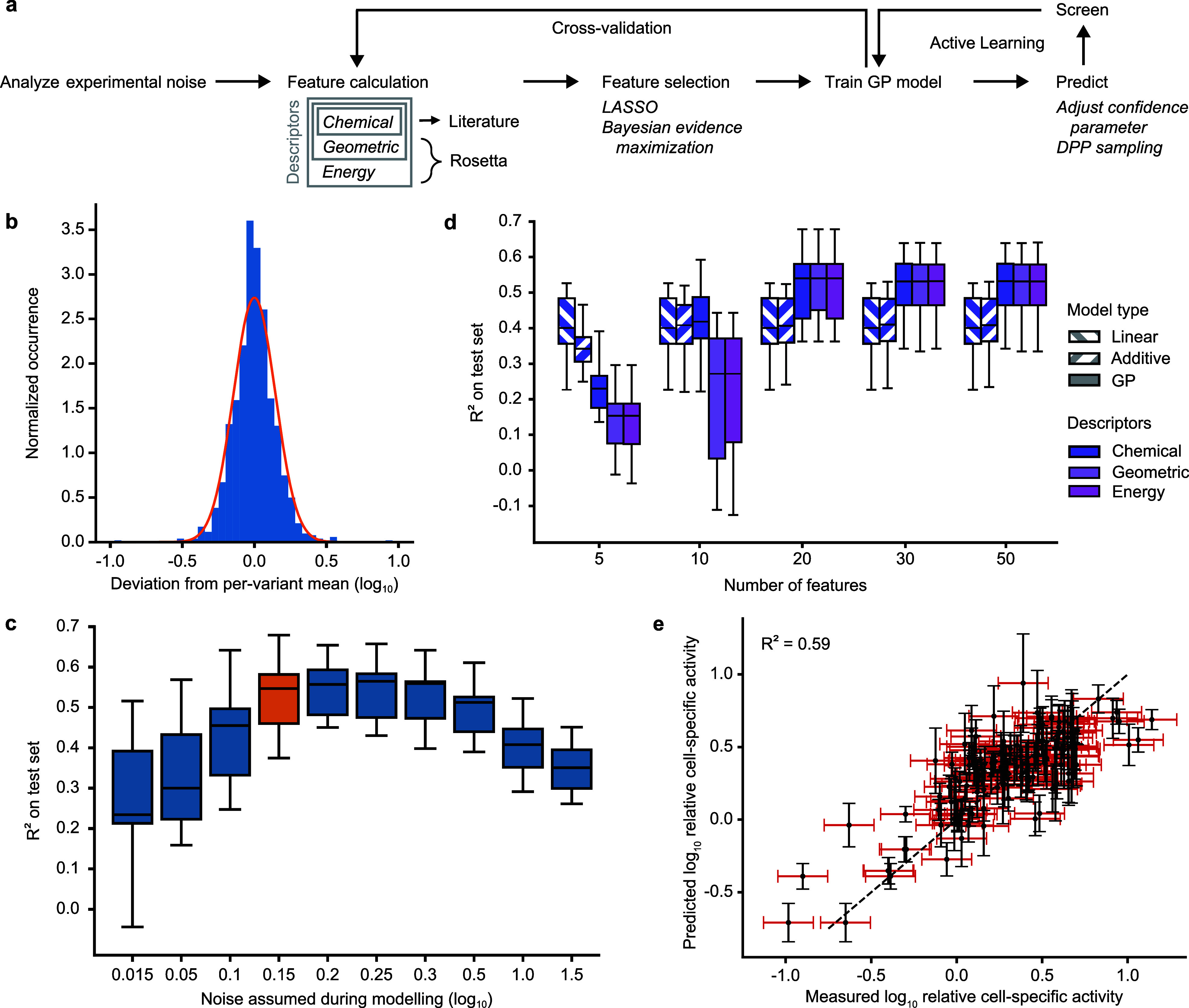
Development of the initial GP model. a. Overview of the machine learning pipeline. Initially, the standard deviation of the activity measurements was estimated to account for experimental noise. Subsequently, three feature sets were calculated and reduced sets were obtained by applying LASSO and Bayesian evidence maximization. The resulting descriptors were then used to train GP models. Model selection and model fitting were benchmarked using cross-validation. Ultimately, the GP model can be used to navigate the sequence space in active learning cycles. b. Histogram of the deviation between replicates in the initial library. The distribution of residuals can be conservatively approximated by a normal distribution with a specific variance (orange). c. Influence of the noise estimate on the predictive performance of the resulting GP model. The value chosen based on Figure 3b is highlighted in orange. The models used here were based on chemical descriptors with 20 features (see Figure 3d) and were evaluated using 15-fold cross-validation. The box plots display the 25^th^, 50^th^, and 75^th^ percentile with whiskers denoting the 1.5-fold interquartile range. d. Influence of feature number (*x*-axis), model type (fill pattern), and descriptors (color) on the performance of machine learning models analyzed by 15-fold cross-validation. The box plots display the 25^th^, 50^th^, and 75^th^ percentile with whiskers denoting the 1.5-fold interquartile range. A comparison of linear models based on different descriptors can be found in Figure S8. e. Performance of the GP model using chemical descriptors and 20 features on an exemplary cross-validation split. The measurement uncertainty (one standard deviation) is displayed in red, while the uncertainty of the model is in black. The R^2^ value of this particular cross-validation split is displayed.

Moreover, our model development pipeline included steps to account for experimental noise and to select suitable descriptors (Figure 3a). Considering the inherent noise in biological experiments during modeling is crucial to ensure that decisions are not influenced by random fluctuations. To distinguish the genuine signal from these fluctuations, it is necessary to define a probabilistic model for data generation, known as the likelihood. This step involves specifying the likelihood and its parameters, which is essential for applying Bayes’ theorem to calculate the posterior distribution (see Methods). To elucidate the form of the likelihood, we relied on the variants appearing multiple times in the screening. This revealed that the deviation of these replicates from the per-variant mean closely follows a log-normal distribution, which can be viewed as a conservative estimate of the experimental noise in the data (Figure 3b). Considering the log-transformed values, this implies a Gaussian likelihood. Next, we used the replicate measurements to determine a standard deviation, which is a key element in defining the data likelihood. We made the simplifying assumption that the variance of the measurement remains constant across the different ArM variants and repeated this analysis after each round of screening. As illustrated in Figure 3c, under- or overestimating the experimental noise leads to a drastically reduced performance of the resulting model, likely due to overfitting to noise in the data. In contrast, the procedure applied here results in a robust performance in the face of noisy data.

With regard to the descriptors that represent the ArM variants during training, we considered features that reflect chemical properties of amino acids^11^ as well as features that were extracted from Sav mutant structures predicted with the Rosetta software.^47^ The latter included both geometric features (e.g., solvent accessible surface area, number of hydrogen bonds, partial charge, dihedral angles, etc.) and energy terms. Note that the geometric descriptors were compiled to be strict supersets of the chemical descriptors (i.e., they also included the chemical descriptors), and similarly the energy-based descriptors are strict supersets of the geometric descriptors. Given the large number of features (125 chemical, 682 geometric, and 161 energy features), we sought to select subsets that are parsimonious while still highly predictive to ensure data efficiency and eliminate redundancy. To this end, we relied on Bayesian evidence maximization (see Methods). Due to the nonlinearity of the optimization challenge, we first reduced the feature sets using LASSO, which performed best in a benchmarking test (Figure S4). More precisely, we fitted a linear model and selected features with nonzero coefficients for automatic relevance detection using Bayesian evidence maximization with a Gaussian process. This allowed us to reduce the initial pool of features to 20–100 and speed up the evidence maximization step, which required multiple optimization restarts to ensure that an adequate maximum was achieved.

Finally, we trained GP models using the different reduced feature sets on the available sequence-activity data and evaluated model performance using 15-fold cross-validation. For comparison, we included a linear and an additive, nonlinear model based on chemical descriptors. The latter is restricted to treating potentially nonlinear effects on the activity additively and is therefore not capable of modeling epistatic effects. Notably, the linear and additive models performed considerably worse than the GP models (Figure 3d), confirming that advanced methods such as GP models are required to accurately capture the sequence-activity relationships in the data. Interestingly, the chemical, geometric, and energy-based descriptors displayed a comparable performance, and a set of 20 features proved to be sufficient in all cases. The most influential features based on automatic relevance detection are listed in Table S1 (see Figure S5 for an analysis of their influence).

As computationally expensive structural calculations are required to generate the geometric and energy-based features and no clear benefit over models relying only on chemical descriptors was observable, we chose to continue with the subset of 20 chemical features as our primary encoding strategy for further modeling. The resulting model displayed a good predictive performance, with a median R^2^ of 0.54 based on 15-fold cross-validation (see Figure 3e and Figure S6 for exemplary validation splits). While leaving room for improvement, this degree of correlation has previously been shown to be suitable for guiding directed evolution campaigns.^11^ Moreover, the median Spearman correlation of 0.68 demonstrates that the relative ranking of variants was largely reproduced by the model (Figure S7), which is important for confident selection of high-activity variants.

### Model Refinement by Active Learning

The aforementioned performance parameters indicate that the initial GP model can predict ArM activity with reasonable accuracy. However, due to the vast sequence space, the random sampling from this space during the generation of training data, as well as inevitable biases in experimental library construction, it is likely that this initial model will not generalize well across the entire sequence-activity landscape. Consequently, it may be “blind” for certain underexplored regions containing highly active ArMs. Therefore, we performed a second, exploratory screening round with the goal of improving the model’s accuracy and ability to generalize across the entire sequence space. To this end, we designed a new library consisting of 720 variants that were primarily selected to be “informative”. Specifically, we utilized the uncertainty estimates of the GP model and selected the variants with the highest uncertainty in the predicted activity among all 3.2 million mutants.^48,49^ This selection was performed in an iterative manner, meaning the uncertainty was recalculated every time after selecting a single variant (see Methods).

We generated these variants based on a pool of oligonucleotides obtained through commercial synthesis on arrays, a method that allows for the cost-efficient construction of large and targeted libraries^50^ and is therefore highly useful for active learning with large batch sizes. After cloning the oligonucleotides into the Sav expression plasmid, we screened the resulting exploration library relying on the automated pipeline in combination with NGS as described above. This exploratory round yielded sequence-activity data on 465 additional variants. It should be noted that this library also contained chimeric variants with amino acid combinations that were not planned in the computational design, likely due to PCR-mediated recombination between variants.^51,52^ While unintended, these additional variants can also be used to augment the machine learning model and were therefore included for training. If desired, chimera formation can be minimized by optimizing the PCR conditions.^51,52^

**Figure 4:**
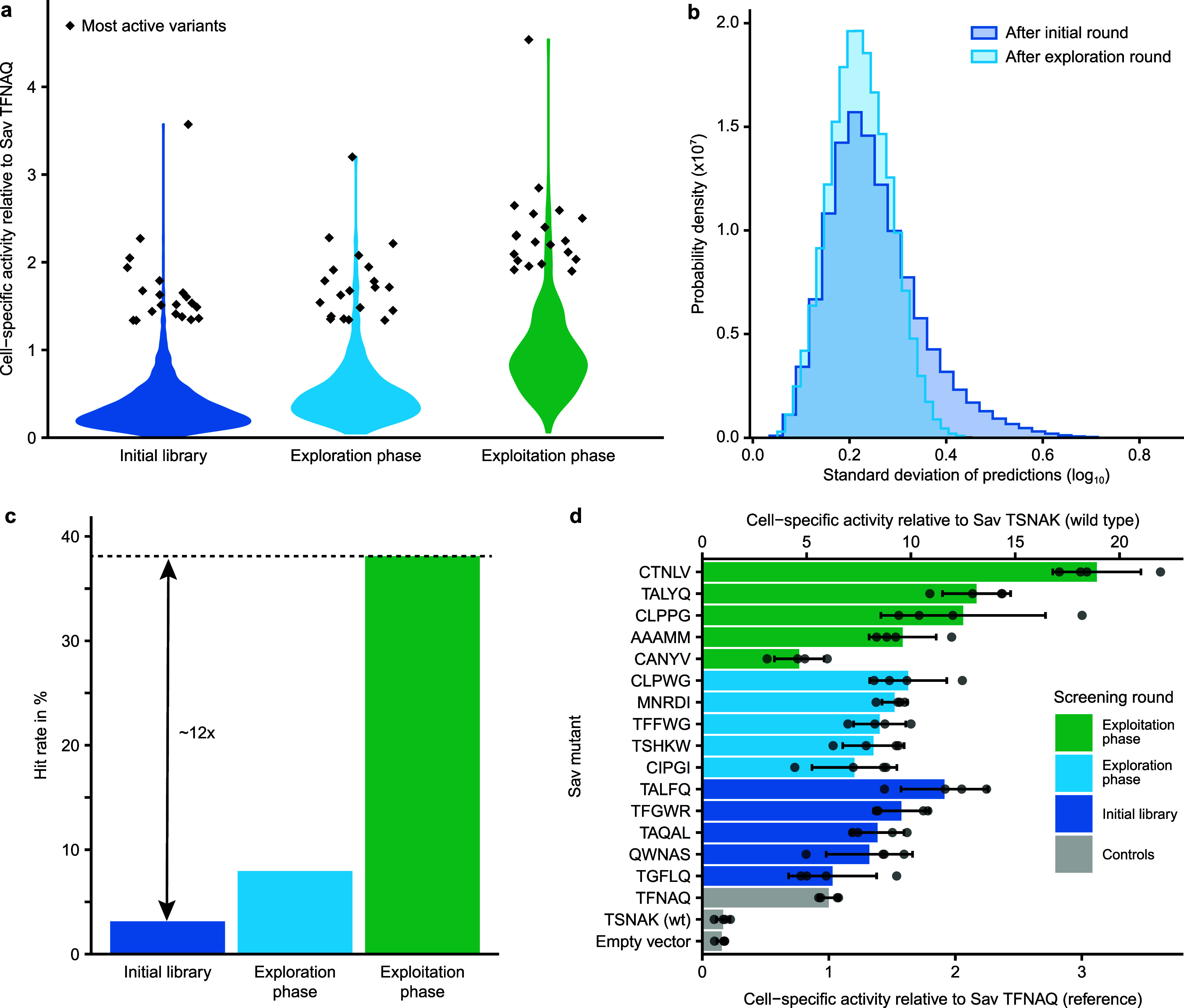
ArM engineering by means of active learning. a. Activity distributions in the three screening rounds displayed as violin plots. The 20 most active variants in each round are depicted as diamonds. Activity is displayed relative to the reference variant (Sav TFNAQ). b. Normalized histograms of the standard deviations of predictions across all 3.2 million variants after the first and second round of screening. c. Hit rate in the three screening rounds. Here, any variant with a higher cell-specific activity than the reference variant is considered a hit. The hit rate represents the fraction of hits among all variants screened in the respective round. Note that the hit rate in the initial library was calculated based on the triple and quadruple mutants, excluding the double mutants that had been tested previously.^40^ In the third round, chimeric variants that were not part of the computationally designed library were excluded to provide a better analysis of the models‘ performance. d. The five most active variants from each screening round were tested again in four replicates. The five-letter codes denote the amino acids in positions 111, 112, 118, 119, and 121 for the respective variants.

The exploration library displayed a similar activity distribution as the initial training data (Figure 4a), which is in line with the focus on informative instead of active variants. Importantly, these new data led to a decrease in the standard deviation of the predictions, most prominently for variants that had previously exhibited a high uncertainty (Figure 4b). While this observation alone is not a proof of increased accuracy, it hints toward an improved representation of previously underexplored regions of the sequence space, which we examined in more detail in subsequent analyses (see below).

### Active Learning Increases the Efficiency of Directed Evolution

Following model refinement in the exploration round, we set out to test whether our model-guided approach can indeed aid in the discovery of active ArMs. With this goal in mind, we designed a third library of 720 variants predicted to be of high activity. Additionally, we employed an in silico diversification step to avoid choosing only variants with highly similar sequences. This provides a safeguard against inaccuracies in the top predictions and increases the likelihood of obtaining variants with diverse properties besides activity (e.g., thermostability, solubility, or activity under alternative conditions). To this end, we used a notion of diversity known as determinantal point processes (DPPs),^48,49^ which use the GP kernel to determine which variants are similar to each other (see Methods and Figure S9a). In short, this approach treats the descriptors of the Sav variants as vectors in Euclidian space and attempts to select a set of vectors that are as orthogonal to each other as possible. We applied this process to a set of variants with the highest predicted activity to obtain a subset of active and yet sequence-diverse variants. This led to a more diverse set of variants compared to a simple greedy selection of the variants with the highest predicted activity as assessed by three different metrics of diversity (Figure S9b). Note that this procedure was not required in the exploration round, as the iterative selection of informative variants naturally leads to a diverse set of mutants.

As described for the exploration round, we obtained the designed library based on an oligonucleotide pool and acquired experimental data for 349 distinct variants. Gratifyingly, this third library displayed a clear shift toward higher activities compared to the first two rounds, both in terms of the average as well as the top activities (Figure 4a). We further analyzed the hit rate in the screening rounds, which we define here as the fraction of ArM variants with higher activity than the reference variant, which is the most active variant identified in a previous study.^40^ While only 3% of the initial library were hits, this rate reached 38% in the exploitation phase, amounting to an approximately 12-fold increase (Figure 4c). This demonstrates that the model acquired a meaningful representation of the activity landscape and can reliably predict active ArMs.

To confirm the results from the different screening rounds, which were performed in single measurements, we tested the most promising variants from all three rounds again in four replicates (Figure 4d). This revealed that Sav 111C 112T 118N 119L 121 V (abbreviated Sav CTNLV) was the most active variant, reaching an 18-fold higher cell-specific hydroamination activity than the wild type (Sav TSNAK) and a 3-fold higher cell-specific activity than the reference variant (Sav TFNAQ). In addition, we purified the most active variants from our whole-cell screening to test whether they also display an increased total turnover number in vitro, which was the case for five of the seven variants tested (Figure S10). As observed before,^40^ the ranking of the variants changed in vitro, which can be expected due to the different reaction environments and varying expression levels in the periplasmic screening.

Notably, the Sav CTNLV mutant does not retain the S112F K121Q mutations that were found to be optimal in the previous double mutant screening.^40^ Likewise, all other variants evaluated in the validation experiment (Figure 4d) retain neither or only one of these two mutations. This highlights the importance of epistatic effects, which can only be adequately considered through combinatorial library designs and nonadditive models. Strikingly, several highly active variants contain a cysteine at position 111, which seems counterintuitive as cysteine has been repeatedly shown to have a pronounced inhibitory effect on gold-catalyzed hydroamination.^53^ However, residue 111 is pointed away from the metal, presumably preventing the thiol from interfering with catalysis. Notably, the beneficial impact of this mutation was not obvious from the initial data set, but became increasingly apparent in subsequent rounds. This indicates that active learning can traverse the mutational space more broadly than alternative methods and enable the identification of counterintuitive effects on activity.

**Figure 5:**
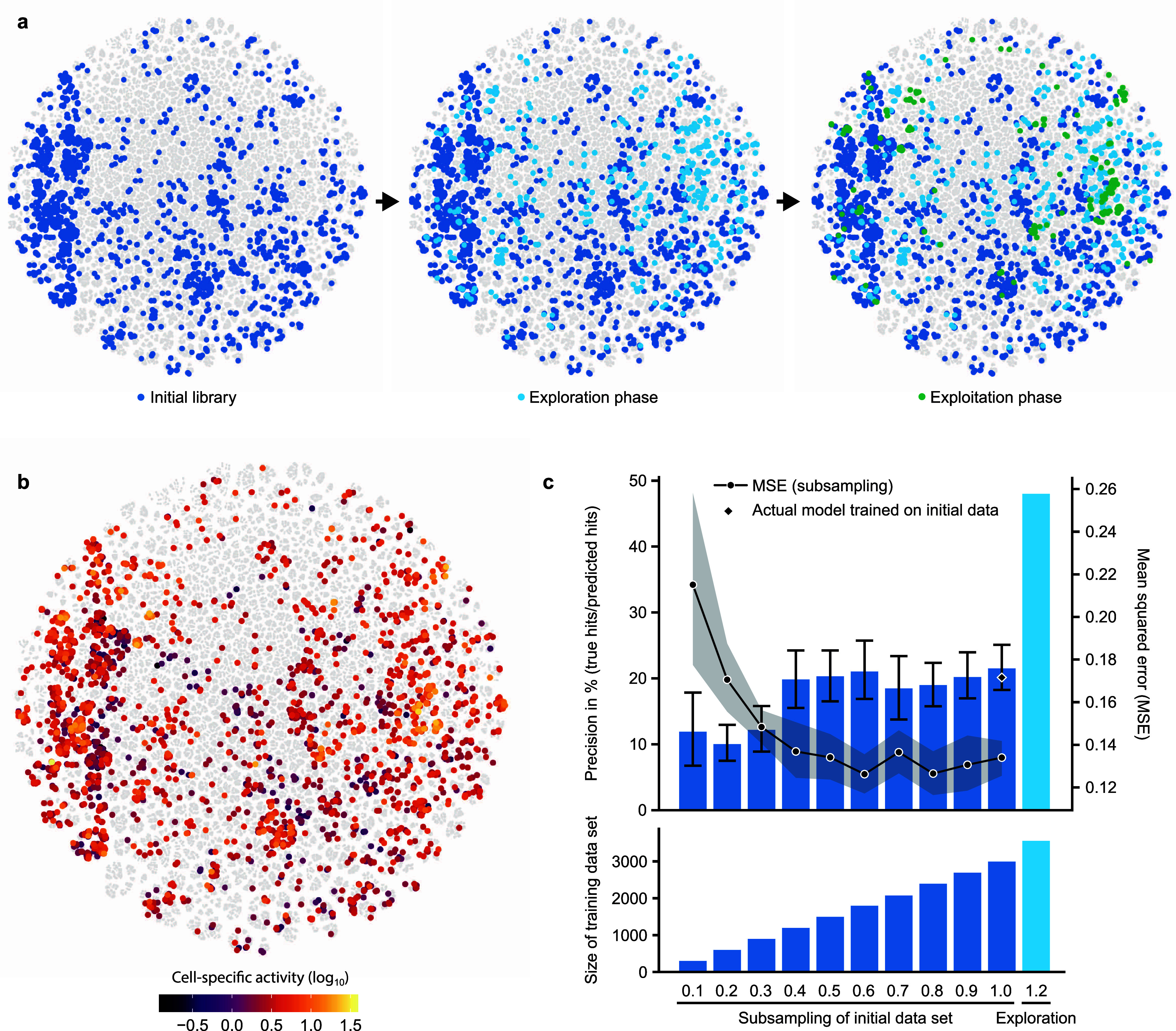
Enhanced sequence-activity mapping through active learning. a. t-SNE visualization of the sequence space. ArM variants that were tested in the three screening rounds are highlighted in different colors. To generate this visualization, all 3.2 million mutants were considered, and a uniform subsample of untested variants was plotted in gray. The similarity metric used was derived from the GP model (see Methods for details). b. t-SNE visualization of the sequence space with color encoding the activity of experimentally tested variants. The clustering is identical to that in Figure 5a. c. Precision in identifying hits and mean squared error (MSE) of predictions as a function of the size of the training data set. The dark-blue bars in the upper graph indicate the average precision of models that were trained on different fractions of the initial data set (screening round 1). The diamond at 1.0 represents the precision of the model used to inform experiments. The light-blue bar on the right represents the model refined by model-guided exploration (screening round 2). Note that the precision is not identical to the experimentally determined hit rate (see Methods). The lower graph depicts the size of the data sets used to train the respective models.

To further corroborate this hypothesis, we performed more detailed analyses to investigate whether the active learning strategy with a model-guided exploration round indeed led to a better representation of the available sequence space. We visualized the sequence space (using t-SNE^54^ on the kernel matrix, see Methods) to analyze how the tested variants are distributed across this space (Figure 5a, b). While care must be taken when interpreting such low-dimensional projections, this analysis indicates that the initial library did indeed not cover the sequence space uniformly. The subsequent exploration round filled in several of the “gaps” in accordance with the design goal of this phase. The exploitation phase focused on a number of regions of high activity, indicating that the selection criteria of high activity and sequence diversity were met. The emergence of multiple clusters of active variants is compatible with the notion of a “rugged” activity landscape with many local optima. Such landscapes can be challenging to navigate using classical methodologies, which frequently follow a single “uphill” trajectory. In contrast, the GP model developed here acquires a holistic understanding of the entire space of 3.2 million ArM variants and allows us to sample various potential optima, increasing the chances of finding suitable variants.

Lastly, we sought to quantify the effect of the applied sampling strategy in relation to the size of the training data set. A crucial question in this regard is whether the active learning strategy suggested here provides a significant benefit over a comparable increase in the size of the training data set by random sampling of variants. To investigate this, we trained models on different fractions of the initial data set using the same model development pipeline as before. As a proxy for an experimentally determined hit rate, we analyzed the models’ precision in identifying hits among the variants tested in the exploitation phase (i.e., the percentage of true hits among variants predicted to be hits). As illustrated in Figure 5c, this analysis indicates that acquiring training data by random sampling is accompanied by strong diminishing returns: Approximately 40% of the initial data set size (equivalent to ∼1200 data points) is sufficient to achieve a similar performance (in terms of precision and mean squared error (MSE)) as a model trained on the entire initial data set (∼3000 data points). This suggests that additional random screening rounds of similar size would not have led to noteworthy improvements of the model. In contrast, the model-guided exploration round, which consisted of only 564 additional data points (an increase of less than 20% in data volume), improved the precision in identifying hits from ∼20% to 48%. This increase is significantly beyond any improvement that can be anticipated due to the mere increase in data volume, emphasizing the fact that this round was substantially more informative than random sampling. This confirms the validity of the suggested active learning and model-guided exploration strategies, pointing to a high potential for enhancing MLDE campaigns while at the same time minimizing the experimental effort.

## Discussion

MLDE is a highly promising strategy for engineering enzymes and other proteins.^1−3,11^ However, the success and efficiency of such engineering campaigns hinges on the ability to generate sufficiently large and informative data sets, the use of smart sampling strategies, and the choice of suitable machine learning techniques that optimally leverage the resulting data.

Many studies on MLDE have relied on small data sets^4−10^ and a single training phase,^4,5,10,55,56^ which may be attributed to experimental limitations. This bears the risk that the resulting models do not accurately represent the sequence space, and thus are likely to leave significant potential hidden within this space untapped. Here, we applied lab automation and NGS to acquire large data sets in a simple and cost-efficient manner, and directed our sampling to the most informative data by means of advanced active learning techniques.

Lab automation greatly increases the throughput of screenings and is, at the same time, highly adaptable to various reactions and target proteins. In this study, we performed some experimental steps manually, but a fully automated workflow could also be implemented. Similarly, the computational pipeline is largely automated, and thus it is conceivable to conduct ArM engineering with minimal human intervention, as was recently demonstrated for the thermostability of a natural enzyme.^57^ Importantly, recent developments such as academic biofoundries and cloud laboratories are making such approaches more widely accessible.^58^

The NGS strategy employed here enables the sequencing of thousands of protein variants for the cost of a small Illumina run and PCR reagents. The former is available for a few hundred dollars (e.g., MiSeq Nano, yielding approximately 1 million reads) and will likely continue to get cheaper. If combined with other samples and run on an instrument with a large capacity, the prorated costs may even be in the range of a few dollars. Regarding the PCR reagents, primer synthesis costs are low as only 20 primers are required to address all 96 positions in a well plate. Similarly, the use of two plate barcodes means that 12 primers for the second PCR are sufficient to distinguish 36 plates. Thus, the required number of primers is lower than in alternative barcoding strategies,^45^ leading to improved scalability. Nonetheless, other methods may be advantageous in specific cases (e.g., when several target genes need to be sequenced). Overall, this workflow enables sequencing at a cost of less than one cent per variant.

Combined, automation and NGS are ideally suited to generate large data sets for MLDE. At the same time, it is also crucial to design information-dense libraries to maximize the efficiency of experimental screening rounds. In the initial round, we achieved this by optimizing the mutational load in the library, which is a straightforward and broadly applicable strategy. Alternatively, zero-shot methods, for example based on ΔΔ*G* calculations,^13^ can be applied as well. In subsequent rounds, library design can be guided by the machine learning model. While it may seem attractive to apply an exploitation-focused strategy to quickly identify active variants, we hypothesized that a model-guided exploration round could substantially improve the predictive performance and thus increase the chances of identifying suitable variants in a subsequent round. Indeed, we observed that the exploration round improved the model’s ability to identify active variants far beyond what would be expected due to the increase in data volume alone. This demonstrates that active learning is a highly effective and efficient strategy for developing accurate models of sequence-activity landscapes. Moreover, the separation into exploration and exploitation phases provides a transparent and practical solution to the exploration-exploitation dilemma, as it allows for a clear and plannable resource allocation. In addition, our study introduces DPP sampling as a strategy for diversifying the selection of active variants, which increases the robustness of MLDE to possible model inaccuracies and may be beneficial with regard to secondary properties beyond activity.

Active learning with large batch sizes, as employed here, may be most attractive when navigating large, rugged sequence-activity landscapes, which is challenging using conventional methodologies. If the screening throughput is limited, for example because of costly reagents or slow analytical procedures, smaller batch sizes can also be used with our methodology. In this case, additional rounds could be performed to increase the predictive performance and the chance of identifying promising variants.

In terms of the machine learning approach, this study corroborates that Gaussian process regression is an attractive choice for MLDE, particularly when strong epistatic effects are present in the sequence-activity landscape. Moreover, it is well-suited for active learning strategies, as the uncertainty quantification is computationally simple, which constitutes an advantage over alternative methods such as deep learning. Our results demonstrate that simple and computationally efficient descriptors are sufficient for nontrivial improvements to engineering campaigns, which is in line with other literature on the subject.^59,60^ Nonetheless, it might be possible to further boost the predictive performance, for example by employing improved structure prediction algorithms or descriptors from modern protein language models.^61,62^ Lastly, our results highlight that accurately accounting for experimental noise is crucial during model development, an aspect that has frequently been neglected.^63^

The application of these strategies to the engineering of ArMs for gold-catalyzed hydroamination led to the identification of a variant with 18-fold higher cell-specific activity than the wild type. Compared to our previous screening of double mutants,^40^ extending the search space to five positions led to a 3-fold improvement. Further rounds of active learning could potentially lead to the discovery of even more active variants. Moreover, the strategies developed here could be used to target additional positions. In this case, minor modifications to the established methods may be required. Most importantly, when engineering more than approximately seven residues simultaneously, the computational search for the most informative or most active variants needs to be restricted (e.g., based on Hamming distance to a parent variant), as exhaustive calculations become impossible due to the exponential increase in the number of possible amino acid combinations. It should be noted that this ArM is likely a challenging engineering target due to the relatively exposed location of the cofactor in Sav. Therefore, applying this engineering strategy to alternative scaffolds with a more shielded active site might enable larger improvements.^64^

Currently, artificial (metallo)enzymes are typically limited by their rather modest activity. Thus, the field could profit greatly from advanced machine learning-guided engineering strategies, as demonstrated here. Similarly, the active learning approach described here could be applied to tailor natural enzymes for industrial applications, or to engineer other proteins such as antibodies, biosensors, or transporters.

## Data Availability

The data and code created as part of this study are available under https://github.com/lasgroup/ml-protein-design-sav-gold.

## Supporting Information Available

Materials and methods, Figures S1–S10 (including additional validation of the pipeline), and Tables S1–S15 (including primer sequences and calculated features) (PDF)
