# Supplementary material for: Enhanced Sequence-Activity Mapping and Evolution of Artificial Metalloenzymes by Active Learning
Source: ACS Cent Sci. 2024 May 22;10(7):1357–70. doi: 10.1021/acscentsci.4c00258 (PMC11273458; doi:10.1021/acscentsci.4c00258)
Supplement: Supplementary file 1 — oc4c00258_si_001.pdf [file oc4c00258_si_001.pdf]

# Supporting Information

# Enhanced Sequence-Activity Mapping and Evolution of Artificial Metalloenzymes by Active Learning

Tobias Vornholt[1,2*], Mojmír Mutný[3*], Gregor W. Schmidt[1], Christian Schellhaas[1], Ryo Tachibana[4], Sven Panke[1,2], Thomas R. Ward[2,4‡], Andreas Krause[3‡], Markus Jeschek[1,5‡]

[1]Department of Biosystems Science and Engineering, ETH Zurich, Mattenstrasse 26, 4058 Basel, Switzerland

[2]National Centre of Competence in Research (NCCR) Molecular Systems Engineering, Switzerland; Web: www.nccr-mse.ch

[3]Department of Computer Science, ETH Zurich, Andreasstrasse 5, 8092 Zurich, Switzerland

[4]Department of Chemistry, University of Basel, Mattenstrasse 24a, 4058 Basel, Switzerland

[5]Institute of Microbiology, University of Regensburg, Universitätsstraße 31, 93053 Regensburg, Germany

* These authors contributed equally

‡ Corresponding authors

## Contents

Materials and Methods ........ 2

Supplementary Figures ........ 10

Supplementary Tables ........ 16

References ........ 22

# Materials and Methods

## Chemicals and reagents

**(Biot-NHC)Au1** was synthesized as previously described[1]. All other chemicals were obtained from Sigma-Aldrich. Primers were synthesized by Sigma-Aldrich, and enzymes for molecular cloning were obtained from New England Biolabs.

## Plasmids

All plasmids were based on a previously described expression plasmid that contains a T7-tagged Sav gene with an N-terminal OmpA signal peptide for export to the periplasm under control of the T7 promoter in a pET30b vector[2]. This plasmid is available from Addgene (#138589). A version of this plasmid encoding the Sav S112F K121Q mutant was used as the starting point for library generation.

## Cloning of Sav libraries

**Site-saturation mutagenesis at 20 positions:** To individually randomize 20 positions in Sav, the plasmid encoding Sav S112F K121Q was amplified in two parts in order to create two overlapping fragments for each position, with mutations being introduced by an NDT codon in one of the primer overhangs. The PCRs were conducted using the primer pairs SSM_X_NDT_fwd and kanR_rev, and kanR_fwd and SSM_X_rev (X denotes the position to be randomized, see Table S2). PCRs were carried out using Q5 High-Fidelity DNA Polymerase (New England Biolabs). Following DpnI digest and PCR purification, the corresponding fragments were assembled by Gibson assembly and transformed into *E. coli* BL21-Gold(DE3). Three clones per position were sequenced by Sanger sequencing to verify correct assembly and diversity at the desired position.

**Double, triple, quadruple, and quintuple mutant libraries:** To generate sets of double, triple, quadruple, and quintuple mutants, the plasmid encoding Sav S112F K121Q was amplified in two parts. One part included the Sav positions 111 and 112, and the other part included positions 118, 119, and 121. To generate fragments with variable but defined numbers of mutations, the primers from Table S3 were used in several PCR reactions according to Table S4. Following DpnI digest and PCR purification, the fragments were assembled in several Gibson assembly reactions as summarized in Table S5. The reactions were then transformed separately into chemocompetent *E. coli* Top10. Plasmids were isolated from the transformants and transformed into the expression strain BL21-Gold(DE3). When picking colonies for screening, the theoretical diversity of the individual sub-libraries (Table S5) was taken into account in order to obtain balanced sets of double, triple, quadruple and quintuple mutants.

**Active learning libraries:** To create libraries of specific Sav variants that were suggested by the machine learning models, oligo pools were ordered from Twist Bioscience. These oligos were used as primers that bind immediately downstream of position 121 in Sav. The 5'-overhang contained the five mutation sites with the desired changes as well as a constant region for Gibson assembly (see Table S6). For the first library of ML-designed variants, insert and backbone were generated according to Table S7. For the second library, the PCRs were run according to Table S8. Following DpnI digest and PCR purification, the fragments were assembled by Gibson assembly and transformed into chemocompetent *E. coli* Top10. Plasmids were isolated from the transformants and transformed into the expression strain BL21-Gold(DE3).

## Sav expression in 96-well plates

96-deep well plates were filled with 500 µL of LB (+ 50 mg $L^{-1}$ kanamycin) per well. Cultures were inoculated from glycerol stocks and grown overnight at 37 °C and 300 revolutions per minute (rpm) in a Kuhner LT-X shaker (50-mm shaking diameter). 20 µL per culture was used to inoculate expression

cultures in 1 mL of LB with kanamycin. These cultures were grown at 37 °C and 300 rpm for 1.5 h. At this point, the plates were placed at room temperature for 20 min, and subsequently, Sav expression was induced by addition of isopropyl-β-D-thiogalactopyranoside (IPTG, final concentration 50 µM). Expression was carried out at 20 °C and 300 rpm for an additional 16 h.

## Whole-cell screening

Following the expression of Sav mutants in deep-well plates, the $OD_{600}$ of the cultures was determined in a plate reader using 50 µL of samples diluted with an equal volume of PBS. Afterwards, the plates were centrifuged (3,220 rcf, 15 °C, 10 min), the supernatant was discarded and the pellets were resuspended in 400 µL of incubation buffer (10 µM **(Biot-NHC)Au1** in 50 mM MES, 0.9 % NaCl, 10 mM diamide, pH 6.1). Cells were incubated with the cofactor for 1 h at 15 °C and 300 rpm. Afterwards, plates were centrifuged (2,000 rcf, 15 °C, 10 min), the supernatant was removed and the pellets were resuspended in 500 µL of washing buffer (50 mM MES, 0.9 % NaCl, 10 mM diamide, pH 6.1). Following another centrifugation step, cell pellets were resuspended in 200 µL of reaction buffer (5 mM 2-ethynylaniline in 50 mM MES, 0.9 % NaCl, 10 mM diamide, pH 6.1). Reactions were performed at 37 °C and 300 rpm for 20 h before determining the product concentration. To account for differences in cell density and plate-to-plate variations, the product concentrations were divided by the $OD_{600}$ of the culture and normalized to the mean of the cell-specific product concentrations measured for the Sav S112F K121Q controls in the respective plate.

## Kovac's assay

Indole was quantified using the photometric Kovac's assay (adapted from Piñero-Fernandez et al.[3]). For measurements in culture supernatant, plates were centrifuged (3,220 rcf, 20 °C, 10 min) and 110 µL supernatant was mixed with 165 µL of Kovac's reagent (50 g $L^{-1}$ 4-(dimethylamino)benzaldehyde, 710 g $L^{-1}$ isoamyl alcohol, 240 g $L^{-1}$ hydrochloric acid) in a separate plate. After 5 min of incubation, these plates were centrifuged (3,220 rcf, 20 °C, 10 min). Subsequently, 75 µL of the upper phase was transferred to a new transparent plate and the absorbance at 540 nm was measured in a plate reader (Tecan Infinite M1000 PRO).

## Lab automation

Colony picking, reaction setup and product quantification were implemented using an automation platform featuring two Tecan EVO 200 (Tecan Group AG) robotic platforms coupled to each other. Both platforms were controlled using the EVOware standard software (Tecan Group AG). Colony picking was performed using the integrated Pickolo system (SciRobotics). For shaking, incubation, and resuspension of cultures, the platform was equipped with a Kuhner ES-X shaking platform (Adolf Kühner AG) running at 300 rpm at 50-mm shaking radius. The shaking platform was surrounded by a custom-made box made of aluminum plastic composite panels (Tecan Group AG). The temperature inside the box was maintained at 15 °C using an "Icecube" (Life imaging services) heater/cooler device. Centrifugation of the samples was performed using the integrated Rotanta 46 RSC Robotic centrifuge (Hettich AG). All buffer exchanges during sample preparation were performed using the integrated liquid-displacement pipetting system equipped with eight 2500 µL dilutors and fixed stainless steel needles. Absorbance measurements were performed using a Tecan Infinite M200 PRO plate reader. The automation method files are available upon request.

## Barcoding of mutants

Following colony picking, cultures were grown overnight at 37 °C and 200 rpm in 96-deep well plates. On the following day, 150 µL per culture was transferred to a 96-well PCR plate. The plates were sealed and placed in a thermal cycler for 5 min at 95 °C to lyse the bacteria. Subsequently, the plates were centrifuged (3200 rcf, 5 min) and 0.5 µL of the supernatant was used as template for the first PCR. This PCR step was done in 96-well plates, with each well containing a distinct combination of barcoded

primers (see Table S9). 30 cycles were performed with 30 s denaturation at 98 °C, 20 s annealing at 71 °C and 30 s elongation by Pfu DNA polymerase at 72 °C. The products from each plate were pooled, run on a 2.5 % (w/v) agarose gel at 100 V for 2 h and purified using a gel extraction kit (Sigma-Aldrich). The products were then used as templates for a second PCR with distinct combinations of barcoded primers (Table S10) to generate a plate-specific labelling. The primer overhangs also contained the adapters required for Illumina sequencing. 30 additional cycles were performed, consisting of 30 s denaturation at 98 °C, 20 s annealing at 63 °C and 30 s elongation by Q5 High-Fidelity DNA Polymerase (New England Biolabs) at 72 °C. Ultimately, all products were pooled, run on a 2.5 % (w/v) agarose gel at 100 V for 2 h, and purified using a gel extraction kit.

## Illumina sequencing

NGS was performed by the Genomics Facility Basel using an Illumina MiSeq platform and a Reagent Kit v2 Nano (150 cycles, PE 110/40) using ~20 % genomic PhiX library as spike-in to increase sequence diversity.

## NGS data analysis

NGS data were analyzed using a custom R script. Forward and reverse reads retrieved from fastq files were paired and target fragments were selected based on several constant regions (GTCACACGTAGCATGTGG, GAGACCTTGTGTCGATGG, GGCCTCGGTGGTGCC, no mismatches). Mutation sites as well as barcodes were extracted based on their distance to these regions. All reads with a Q-score < 30 at the mutation sites were discarded, as well as those for which a barcode did not match any of the expected sequences. The codons at the mutation sites were translated to amino acids in order to identify the Sav variants and the barcodes were used to identify the plate and well for each read. For each plate, the entries were then grouped by variant and only the combinations of variant and well with the highest number of reads was kept. This eliminates combinations of variants and barcodes that result from chimera formation during the second PCR step. Subsequently, variants that accounted for less than 80 % of reads for a given barcode combination were discarded in order to eliminate cases where more than one variant had been present in a well.

## Sav expression for purification

A single colony of *E. coli* BL21-Gold(DE3) harbouring a plasmid for periplasmic expression of the desired Sav variant was used to inoculate a starter culture (4 mL of LB with 50 mg $L^{-1}$ kanamycin), which was grown overnight at 37 °C and 200 rpm. On the following day, 100 mL of LB with kanamycin in a 500 mL flask was inoculated to an $OD_{600}$ of 0.01. The culture was grown at 37 °C and 200 rpm until it reached an $OD_{600}$ of 0.5. At this point, the flask was placed at room temperature for 20 min and 50 µM IPTG (final concentration) was added to induce Sav expression. Expression was performed at 20 °C and 200 rpm overnight, and cells were harvested by centrifugation (3,220 rcf, 4 °C, 15 min). Pellets were stored at -20 °C until purification.

## Sav purification

Cell pellets were resuspended in 10 mL of lysis buffer (50 mM tris, 150 mM NaCl, 1 g $L^{-1}$ lysozyme, pH 7.4). After 30 min of incubation at room temperature, cell suspensions were subjected to three freeze-thaw cycles. Subsequently, nucleic acids were digested by addition of 10 µL of DNaseI (2000 units/mL, New England Biolabs) and $CaCl_2$ to a final concentration of 10 mM, followed by incubation at 37 °C for 45 min. After centrifugation, the supernatant was transferred to a new tube and mixed with 40 mL of binding buffer (50 mM ammonium bicarbonate, 500 mM NaCl, pH 11). Pierce iminobiotin agarose (Thermo Fisher Scientific) was equilibrated in falcon tubes and used to pack a PD-10 column up to a bed height of approximately 1 cm. The lysate was loaded onto the column relying on gravity flow. Subsequently, the column was washed twice with 10 mL binding buffer. Ultimately, Sav was eluted using 10 mL of elution buffer (50 mM ammonium acetate, 500 mM NaCl, pH 4). Amicon Ultra

filters (10 kDa molecular weight cut-off) were then used to concentrate the samples and exchange the buffer against the reaction buffer (50 mM MES, 0.9 % NaCl, pH 6.1).

## Quantification of biotin-binding sites

The concentration of Sav biotin-binding sites was determined using a modified version of the assay described by Kada et al.[4], which relies on the quenching of the fluorescence of a biotinylated fluorophore upon binding to Sav. Specifically, 190 µL of the binding site buffer (1 µM biotin-4-fluorescein, 0.1 g $L^{-1}$ bovine serum albumin in PBS) was mixed with 10 µL of purified Sav. After incubation at room temperature for 90 min, the fluorescence intensity was measured (excitation at 485 nm, emission at 525 nm), and a calibration curve produced with lyophilized Sav was used to calculate the concentration of Sav biotin-binding sites.

## *In vitro* catalysis

*In vitro* reactions were performed with 2.5 µM purified Sav (tetrameric; corresponding to 10 µM biotin-binding sites), 5 µM **(Biot-NHC)Au1** and 5 mM 2-ethynylaniline in MES buffer (50 mM MES, 0.9 % NaCl, pH 6.1). The reactions were performed in a volume of 200 µL in glass vials and were incubated at 37 °C and 200 rpm for 20 h. Subsequently, the indole concentration was determined using the Kovac's assay.

## Machine learning

All machine learning methods were implemented in Python using scikit-learn[5], Pytorch[6], Biotite[7], pyRosetta[8] and SciPy[9].

**Calculation of descriptors:** In this work, we encoded the Sav mutants by three different classes of descriptors: chemical descriptors, geometric descriptors, and energy-based descriptors. To obtain the chemical descriptors, we utilized amino-acid descriptors from four different sources: Z-scores[10], VSHE[11], Barley score[12], and PCscores[13]. All of these are based on physical amino-acid properties (see Table S11) and principal component analysis (PCA) was used to construct a reduced representation. Here, we concatenated these features, resulting in 25 values per amino-acid position. As we considered quintuple mutants, each Sav variant is thus described by 125 features.

The geometric and energy-based features were created using the Rosetta software. First, we calculated the approximate dimeric structure of each mutant with a fixed seed using the *mutate* function with the default distance for post-mutational changes. The mutations were performed in the order of the five sites in the primary protein sequence (111, 112, 118, 119, 121). We calculated all 3.2 million approximate Sav dimer structures. Next, we used the package Biotite to calculate charge, distance to the centre of mass, and radii of each amino-acid residue. Additionally, we calculated the solvent accessible surface area of each residue, the number of hydrogen bonds per residue, and the dihedral angles. A summary of the features can be found in Table S12. We discarded variables that did not vary across the 3.2 million structures, leaving us with 682 features. The energy-based features were calculated in the same manner as the geometric features using the approximate structure of the variant and correspond to the ref2015 set of 31 features per mutant from the Rosetta suite (see Table S13). A common pre-processing step applied to all features involved subtracting the mean of each descriptor across the 3.2 million variants and scaling by the absolute value of the maximum value of that descriptor. This process ensured that the descriptors fell within the range [-1,1] and that their average value was zero.

**Likelihood elucidation**: The first step of any data analysis is to understand its randomness and generation process. In our case, the likelihood specified the experimental error introduced by biological variability, the measurement procedure, etc. In other words, we assumed that our measurements were corrupted by additive noise under log transformation. To justify this hypothesis, we analysed the distribution of the differences between replicates from their mean value. As a normal

distribution appeared to be a good and conservative approximation for these data, we used a Gaussian likelihood with a standard deviation determined from the aforementioned distribution. In the first round, this value was determined to be 0.15, rounded to two decimal points in the log-transformed cell-specific activity. We repeated the same procedure for the subsequent screening rounds to account for variability between experiments. The standard deviations determined for the second and third round were 0.20 and 0.12, respectively.

**Model section:** For further analysis and Gaussian process fitting, we did not use the full set of features due to the complexity of the initial fitting procedure, which involves optimizing the marginal likelihood[14]. To simplify this process, we preprocessed the initial set of descriptors using one of three straightforward machine learning models: LASSO, elastic net, and random forests. We evaluated the effectiveness of this procedure through cross-validation on the entire feature space. In all cases, we utilized the scikit-learn implementation of these methods. Both the LASSO and elastic net methods employed an adaptive selection of the regularization parameter, which involved an additional layer of cross-validation within the training split. For random forests, we used a configuration of 500 trees with a maximum depth of 15 and a minimum split size of 5. After training, we selected *k* descriptors with either the largest coefficients or the highest feature importance for further analysis. We varied *k* across 20, 40, 60, 80, and 100. This range was chosen as the maximum set of descriptors that we believed would allow the Gaussian process library to reliably optimize the marginal likelihood.

**Gaussian process:** The functional relationship between the Sav sequence and ArM activity was modelled using Gaussian processes (GPs). This Bayesian method is versatile in capturing a wide range of structures, and is defined by its mean and covariance function, also known as the kernel. In our case, we found that kernels of the following form performed best among selected statistical models with calibrated uncertainty:

$$\mathrm{k}(p,\tilde{p}) = \kappa(poly(d(p,\tilde{p})))\exp(-d(p,\tilde{p})^2)\ where\ \ d_\gamma(p,\tilde{p}) \propto \left(\sqrt{\sum_{j=1}^{m} 1/\gamma_j^2\left(\Phi_j(p)-\Phi_j(\tilde{p})\right)^2}\right).$$

This kernel is known as Matérn kernel with regularity parameter η=5/2 and is commonly used to model twice differentiable smooth response surfaces[15]. The letters p and p' denote different protein variants of which we want to calculate similarity. The function $\Phi$ corresponds to the feature representation of the protein p. In this work, this is a function that maps the protein sequence or structure to a fixed length vector. The parameters $\gamma_i$ are usually referred to as length scales and are used for automatic relevance detection[16]. They guide the importance of a certain variable, i.e., if γ is very large, this part of the descriptor vector $\Phi$ has less impact if changed than a coordinate $\Phi_j$ with larger $\gamma_j$. The length scales can be selected based on Bayesian evidence maximization, which is a well-tested methodology to select length scales that most likely explain the activity data[14]. The parameter κ was selected using the expected maximal achievable improvement of the protein, in this case $\kappa = 3$, meaning that the maximum achievable improvement is 1000-fold over the wild-type variant (due to modelling $\log_{10}$).

**Bayesian evidence maximization:** Hyperparameters, specifically the length scales of the Matérn kernel, were optimized for each of the chosen features using the maximization of evidence, a common Bayesian approach[15]. As before, we denote length scales $\gamma$. By maximization of evidence, we mean

$\gamma^* = argmax_\gamma\, P(D|\gamma)$ and $P(D|\gamma) = \int P(D|f,\gamma)P(f,\gamma)df,$

where $P(f|\gamma)$ is the Gaussian process prior parametrized by length scales, and $P(D|f,\gamma)$ is the Gaussian likelihood as specified in the prior section on likelihood elucidation. The integration in the prior formula represents marginalization of the Gaussian process, and strictly speaking integration

requires certain mathematical regularity conditions, which we omit here. Upon finding the right length scales from the initial data, these were fixed, and the posterior $P(f|\gamma, D)$ was calculated after each experimentation round without changing them. To implement the Bayesian posterior calculation, we used a custom implementation in Python.

## Active Learning

To employ active learning, we used a technique similar to the upper confidence bound method as described by Srinivas et al.[17], or greedy information maximization. In the exploration round, we generated predictions using the GP model based on chemical descriptors with 20 features. To select informative variants, the confidence parameter was set to infinity. The selection was performed in an iterative manner: After selecting the variant with the highest uncertainty and treating it as a new measurement, the model was retrained, the predictions for the remaining variants were updated, and the mutant with the highest uncertainty among these new predictions was selected next. This approach is possible as the uncertainty is independent of the measurement value in the model used here. This procedure has a mathematical relation to the DPP sampling applied in the exploitation round and ensures that a sequence-diverse set of informative variants is selected. In addition, we allocated a smaller part of the experimental budget to variants predicted to be active to validate the initial model. The latter budget was split equally into three categories: A conservative set representing the Sav mutants for which the mean prediction minus two standard deviations was highest, as well as balanced and optimistic predictions chosen based on the mean and the mean plus two standard deviations as ranking mechanisms, respectively. See Table S14 for an overview of the budget allocation in the exploration round. We obtained additional data points through a small random mutagenesis as well as chimeric variants, which were not part of the designed library.

In the exploitation round, we aimed to select active and diverse ArM variants. To this end, we trained three GP models on the new data set (including the exploration round). The three models employed different descriptors (chemical descriptors with 20 features, geometric and energy –based descriptors with 50 features) to possibly obtain more diverse predictions. We split the experimental budget equally among the three models. Further, we split the experimental budget per model into conservative and balanced predictions (see above). The experimental budget allocation can be found in Table S15. The confidence parameter was set to 2 for the exploitation round. Additionally, a diversifying principle based on determinantal point processes[18], a mathematical model of diversity, was employed to choose a diverse subset of variants, following the principles described by Nava et al.[19] (see below). Upon retrieval of the above budget, we performed a validation step. As part of it, we augmented the chemical descriptor model with the new data and proposed 30 additional Sav variants to test for potential improvements. These were selected to be conservative or balanced (10 variants each), and 10 variants were selected to be the best predicted according to the balanced prediction metric.

## DPP sampling

When selecting Sav variants for experimental testing, it is advisable that these are diverse, especially in the context of the exploitation round. For example, if we were to identify the best *x* candidates using the machine learning pipeline, it is very likely that all these top *x* candidates are highly similar to each other for small *x*. If the model happens to be incorrect with regard to the top predictions, this will lead to failure to identify any active mutants. A more principled approach is to pick a diverse subset. Namely, select a set of promising mutants, and then further select a subset of these which is diverse. This ensures robustness to potential misspecification errors. The model of diversity we employed here is the inverse of the similarity model we used to train the GP regressor, namely the kernel. We measured the diversity of the selected subset by the determinant of the kernel matrix. This is a common approach in the machine learning literature[18], as it has an intuitive interpretation where the

determinant between two vectors is proportional to the volume that the two vectors span (Figure S7a). The more orthogonal (dissimilar) these two vectors are, the larger the volume. A natural extension to non-parametric models such as GP models is to use the kernel matrix instead of the inner product between vectors. Finding a subset of maximum determinant is an NP-hard problem[20]. Hence, often a probabilistic method is employed to find the subsets[19,21].

Suppose that the probability of sampling a set is proportional to the value of the determinant for this set. This probabilistic object is known as determinantal point process (DPP)[18] and can be sampled very efficiently. In order to diversify our top-*x* batches, we select a top *y* number of candidates, where *y* is bigger than *x*, from which we choose a diverse set of size *x* using DPP sampling. The value of $y = 500$ was chosen arbitrarily for our experiments. The value of *x* depends on the available experimental budget in each round. The explorative round does not require diversification as the goal to select informative Sav variants already leads to diversity. In fact, it is related to the greedy search for a set with the largest determinant[21].

In order to compare the diversity of the measurements, we use the isometry score, which is a ratio determinant and trace of a kernel matrix defined via the batch of sequences. The score equates to the normalized ratio of trace and determinant.

$$I(K) = \left( \frac{det(K)^{\frac{1}{n}}}{\frac{1}{n} trace(K)} \right).$$

The score is valued between 0 and 1, where 1 is achieved once K forms essentially a diagonal matrix. If this is the case, this means the implicit features (defined via the kernel) are orthogonal to each other. On the other hand, 0 indicates that the implicit features defined via the kernel are very closely aligned to each other. Of course, this score depends on the kernel metric we use. The DPP method practically maximizes this metric under the models' kernel in expectation.

## Clustering of ArM variants

The clustering shown in Fig. 5 was created using the t-SNE (t-distributed stochastic neighbour embedding)[22] clustering methodology. For this analysis, we used the kernel matrix of the chemical descriptor model. This model is based on a Gaussian process with ARD (automatic relevance determination) kernel length scales. The t-SNE algorithm clusters the data based on a similarity metric that includes exponentiated negative Euclidean distances. This is very similar to our machine learning model, with the exception that instead of a pure exponential, we use the Matérn kernel. However, this should qualitatively lead to similar results. Hence, to generate the clustering, we took the chemical descriptors, scaled them with appropriate length scales, and used the scikit-learn implementation of the t-SNE algorithm to generate the clusters. We tested several values of complexity, and the plotted clusterings correspond to a value of 150, as it appeared to generate the most structured results.

## Subsampling analysis

To analyse the effect of data set size on the predictive ability of the model, we created 20 random subsamples of the original data set for each subsampling fraction (0.1 - 1 in intervals of 0.1). We then applied the previously described machine learning pipeline, starting with the feature selection. To analyse the performance of the models, we used them to predict the activity of all ArM variants that were tested in the exploitation round, and calculated the mean squared error of the predictions as well as the precision in predicting hits (i.e., ArM variants with a higher activity than the reference variant). Precision is defined as the percentage of true hits among predicted hits. To investigate the effect of the exploration round, we calculated the precision of a model that was trained on all data from the initial library and the exploration round. In the latter case, the precision is different from the

experimentally determined hit rate as not all experimentally tested variants were predicted to be hits by the model used here.

# Supplementary Figures

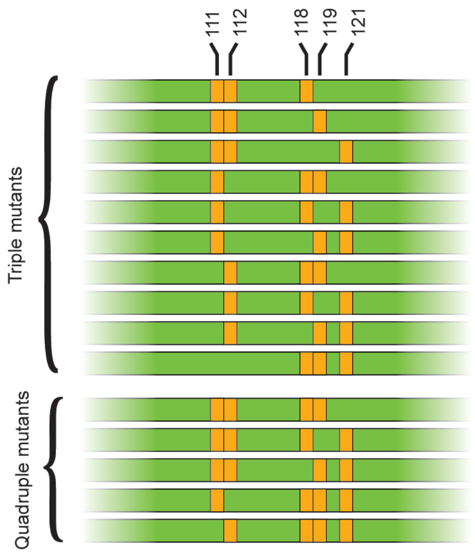


**Figure S1. Illustration of the library design comprising both triple and quadruple mutant sub-libraries.** The orange rectangles indicate randomized positions in the Sav gene. When keeping two or one out of five positions constant, it is possible to create 10 sets of triple mutants and 5 sets of quadruple mutants. Note that the reference variant Sav S112F K121Q was selected as the parent of this library.

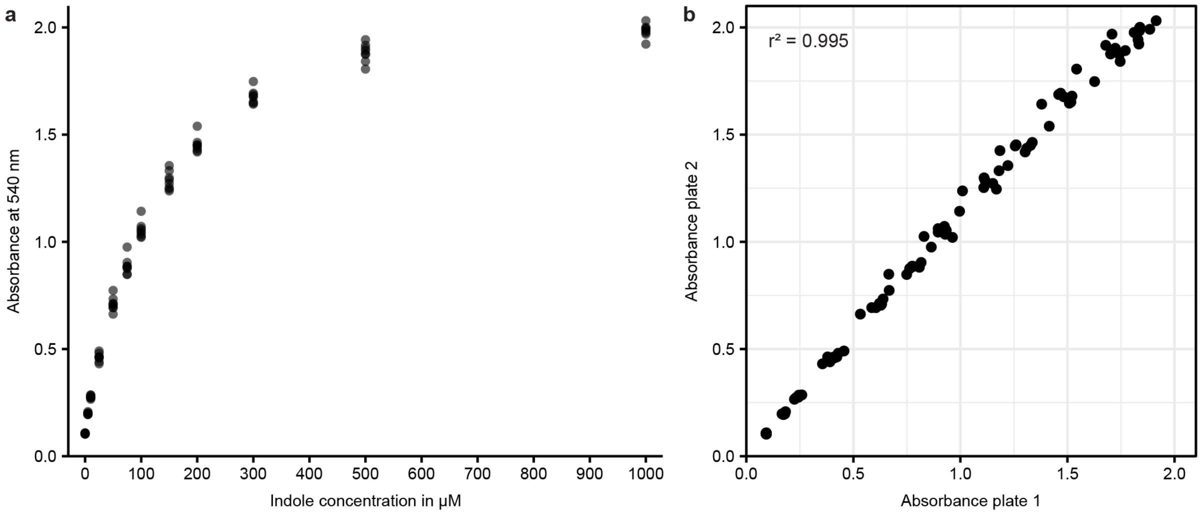


**Figure S2. Validation of the automated Kovac's assay. a**, Indole standard curve measured using the automated Kovac's assay. Eight replicates were measured per concentration and all samples were in the same 96-well plate. Note that the indole concentrations observed in screenings of ArM mutants are below 100 µM. **b**, Reproducibility of indole measurements using the automated Kovac's assay. Two 96-well plates were filled with identical indole standards and subjected to the automated assay. The absorbance values of corresponding samples are plotted against each other and show a high correlation as determined by linear regression.

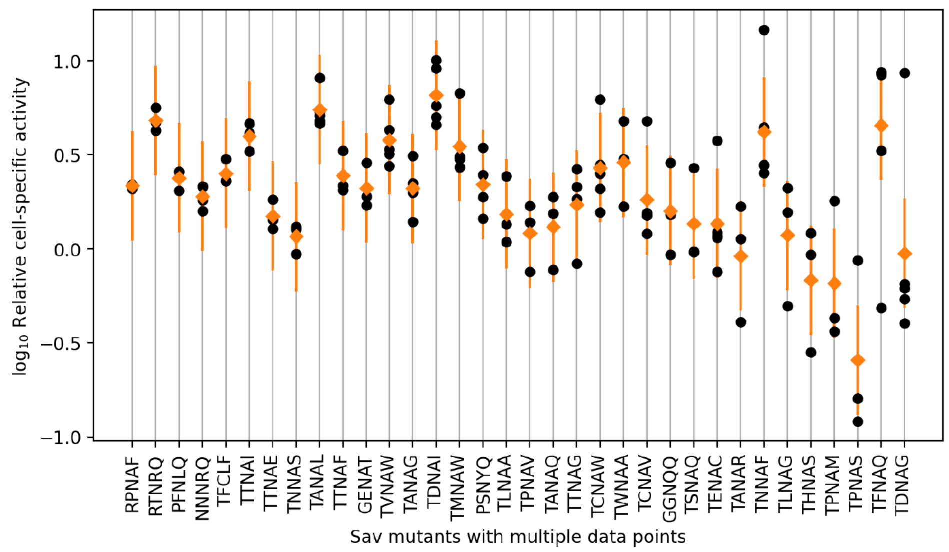


**Figure S3. Reproducibility in the initial screening.** Points on the vertical lines correspond to variants that were found more than twice in the library. The variance between these replicates increases from left to right. The orange diamonds indicate the mean of the replicates and the orange lines display the two-fold standard deviation.

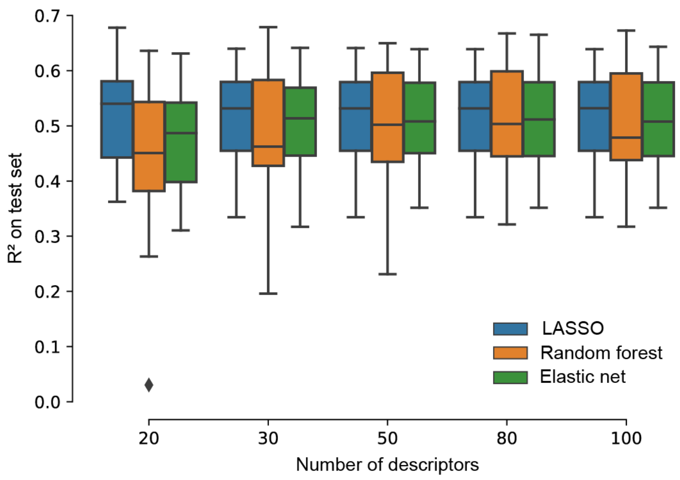


**Figure S4. Benchmarking of the feature selection mechanism to create a subset for the final Bayesian model selection with automatic relevance detection.** We benchmarked three methods: two linear methods (LASSO and elastic net), and one non-linear method (random forest using its feature relevance score). LASSO generally performed best, particularly in selecting 20 features, likely due to its simplicity and robustness against noise. All methods were implemented using the scikit-learn toolkit. The benchmarking was done using 15-fold cross validation making sure no duplicates are shared between test and train.

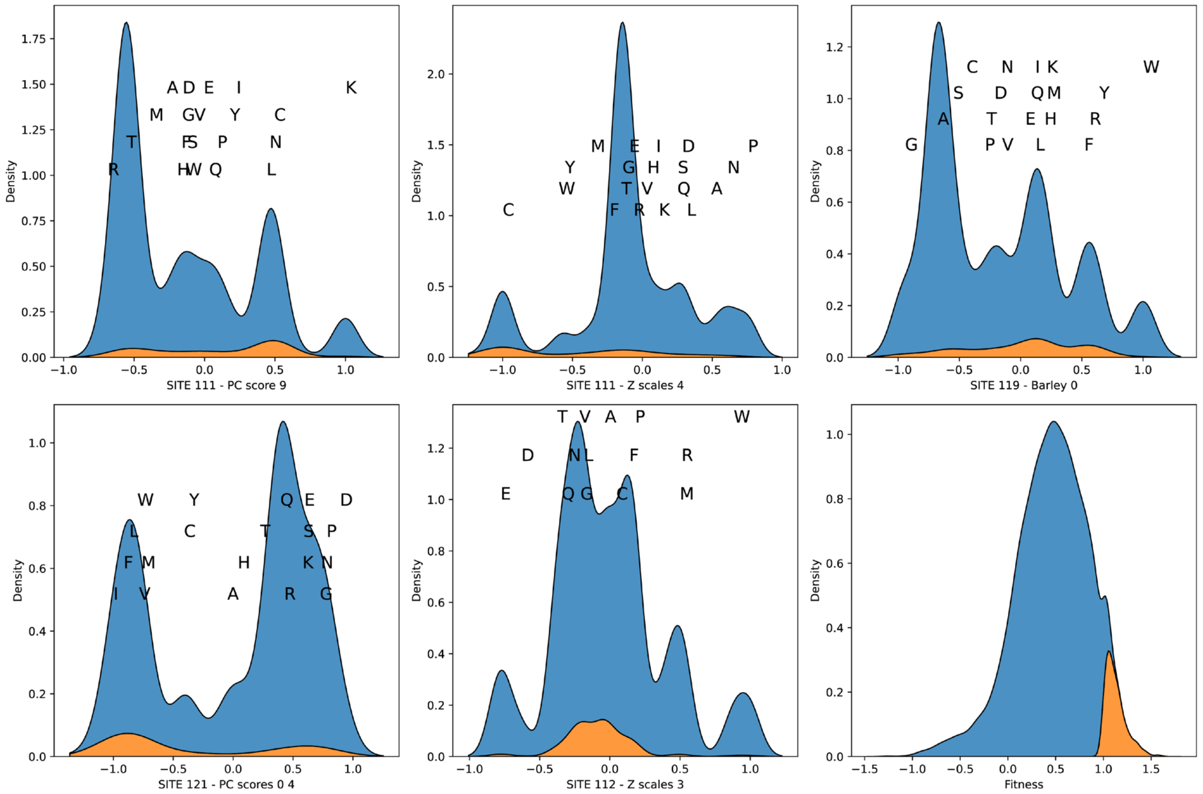


**Figure S5. Distribution of the five most important features in the data set.** The blue area represents the background distribution, while the orange highlights the distribution of variants that are more active than the reference variant Sav TFNAQ. Notably, the orange distribution sometimes appears as a single peak and other times as bimodal, suggesting multiple ways of achieving high activity. The characters mark the value of the given feature that corresponds to the respective amino acid. The plots also demonstrate that different amino acids at specific sites can result in identical feature values. For example, as shown in the upper-left panel, amino acids D, G, F, and H all yield the same feature value. The bottom-right panel displays the distribution of activity values. The names of the features are given in the x-label.

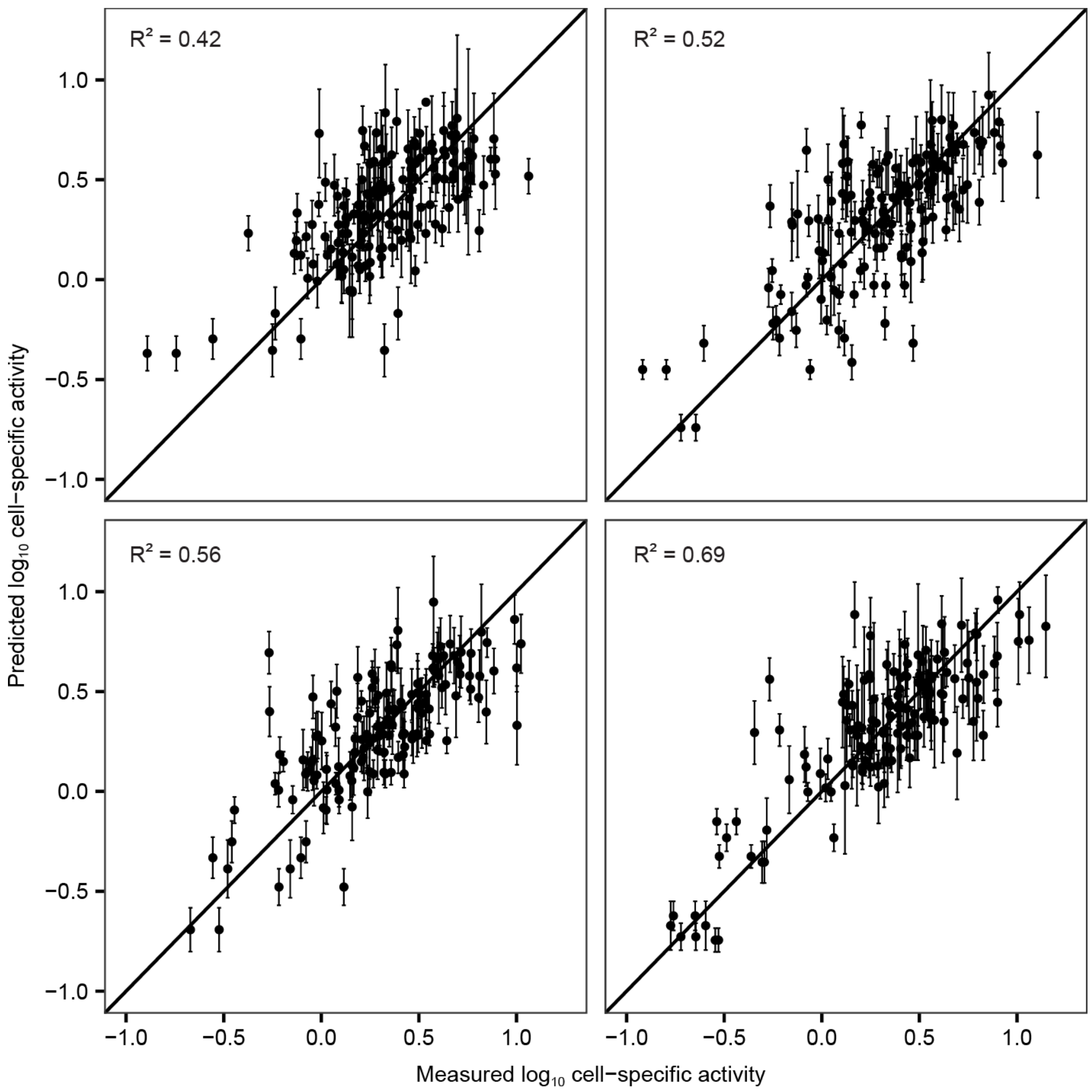


**Figure S6. Exemplary cross-validation splits from the initial training phase.** The underlying model is the GP model using chemical descriptors with 20 features. The top left graph depicts the validation split with the lowest correlation, while the bottom right graph shows the split with the highest correlation. The other plots show splits with an intermediate correlation.

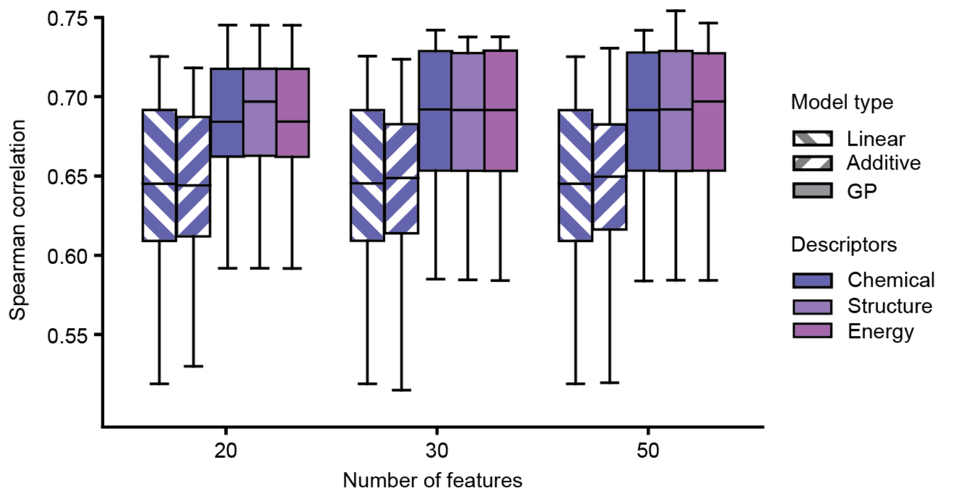


**Figure S7. Spearman correlation (based on 15-fold cross-validation) of several models trained on the initial data set.** The influence of the number of features, model type, and descriptors was investigated.

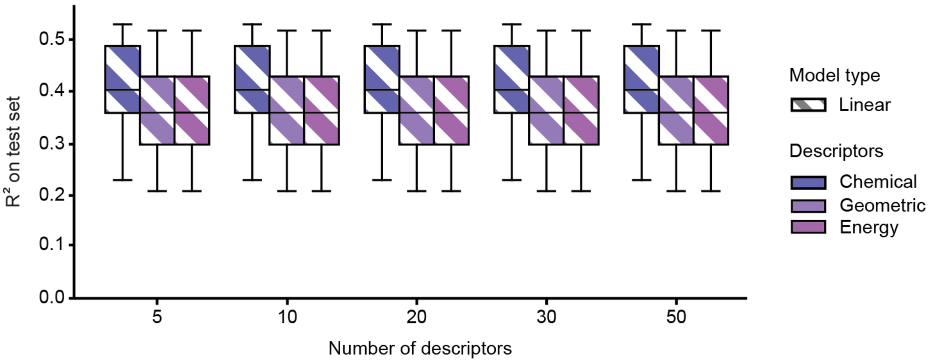


**Figure S8. Performance of linear models based on different descriptors analysed by 15-fold cross-validation.** The box plots display the 25th, 50th and 75th percentile with whiskers denoting the 1.5-fold interquartile range. This figure is an extension to Figure 3d.

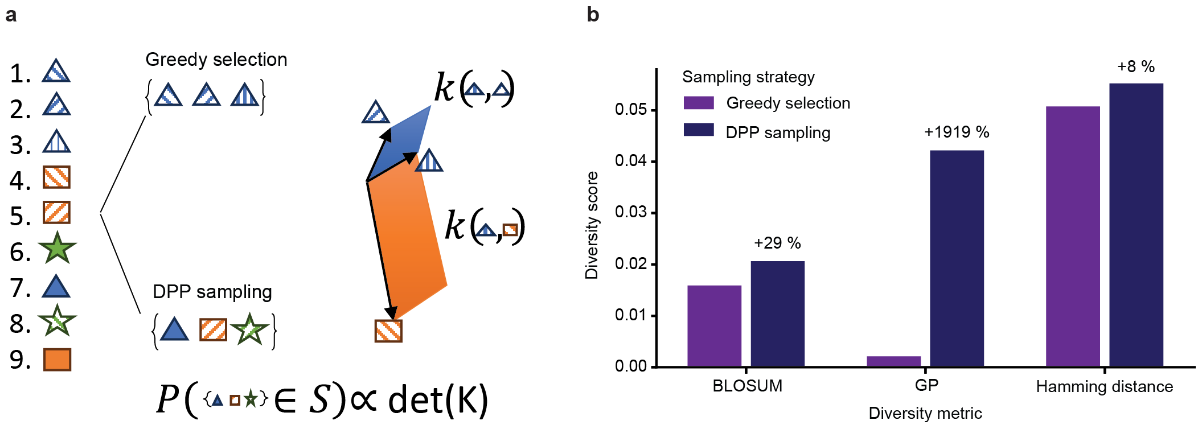


**Figure S9. Diversification using determinantal point processes (DPP). a**, Illustration of DPP sampling. A ranked set of variants is depicted as pictograms. The same colour and shape indicate similar enzymes. Whereas greedy selection of top-ranked variants is likely to result in a set of highly similar variants, DPP sampling selects a more diverse batch (left). DPP sampling picks candidates based on the volume that their representations span in Euclidian space (right). More different variants span a larger volume, hence there is a larger probability of them being selected. An idealized batch would be such that all vectors are orthogonal to each other, forming a hypercube. **b**, Comparison of two sampling strategies in the exploitation round: Diversified selection based on DPP and greedy selection of the most active variants (predicted by the model using chemical descriptors). The diversity of the two sets of variants was compared using three metrics: BLOSUM90 substitution matrix, the model-based metric (GP), and the Hamming distance, sometimes also referred to as one-hot distance. Note that the different metrics are only expected to correlate weakly with each other. As the GP model was used to make the selection, the considerable increase in diversity according to this metric is expected.

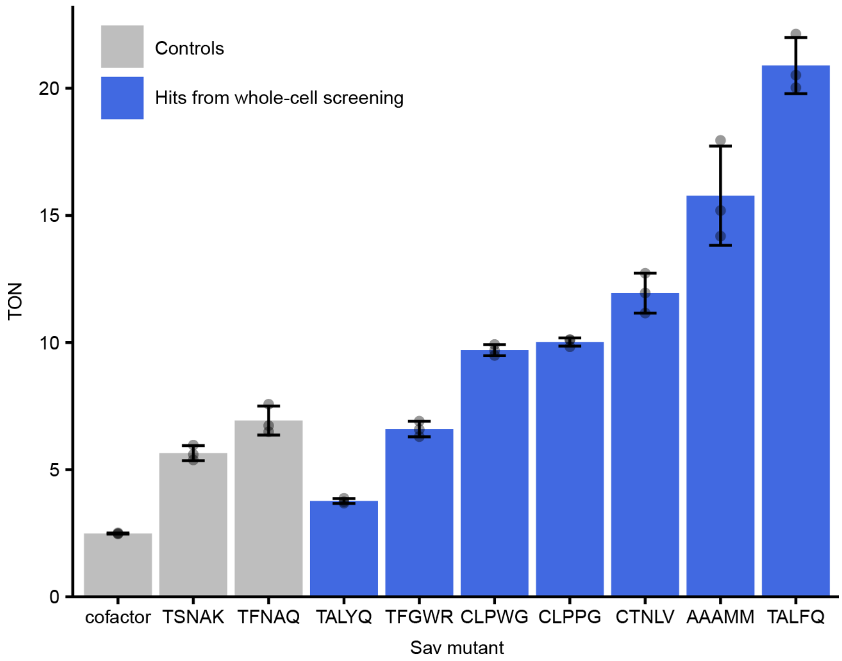


**Figure S10.** ***In vitro*** **turnover number (TON) of ArM variants identified in the whole-cell screening.** The Sav variants with the highest cell-specific activity for hydroamination according to the validation experiment (Fig. 4d) were purified. Reactions were performed in triplicate with 10 µM Sav biotin-binding sites, 5 µM **(Biot-NHC)Au1** and 5 mM 2-ethynylaniline. For comparison, reactions were also performed using the free cofactor without Sav. After 24 h, the indole concentration was determined using the Kovac's assay. TSNAK is the wild-type variant, and TFNAQ refers to the best variant identified in a previous screening. Note that the TONs determined here are lower than in previous studies, possibly due to a less active batch of **(Biot-NHC)Au1**.

# Supplementary Tables

**Table S1. Most informative features identified by automatic relevance detection for Gaussian processes.** Most features are not interpretable, except for the Barley score which correlates with the size of the amino acid at position 119. When we overlay the distribution of the active vs. non-active variants, we discover that these features for active variants follow three different patterns. Optimal – meaning all good variants have this feature corresponding to one value, approximately bimodal – there are roughly two good values for these features, or strongly-bimodal – representing two equally good options for this feature. A visual depiction of this analysis can be found in Figure S5.

| Feature | Site | Interpretation | Effect |
|---|---|---|---|
| PC 9 | 111 | Chemical | Approximately Bimodal |
| Z-score 4 | 111 | Chemical | Approximately Bimodal |
| Barley 0 | 119 | Chemical (size) | Tri-modal |
| PC 0 | 121 | Chemical | Strongly Bimodal |
| Z-score 3 | 121 | Chemical | Optimal |

**Table S2. Primers used for site-saturation mutagenesis at 20 Sav positions in proximity to the cofactor.** Mutations were introduced using NDT codons, which are highlighted in bold.

| Name | Sequence (5' → 3') |
|---|---|
| SSM_47_NDT_fwd | TGACCGGAACCTACGAGTCGGCC**NDT**GGCAACGCCGAGAGC |
| SSM_48_NDT_fwd | CCGGAACCTACGAGTCGGCCGTC**NDT**AACGCCGAGAGCCGC |
| SSM_49_NDT_fwd | GAACCTACGAGTCGGCCGTCGGC**NDT**GCCGAGAGCCGCTAC |
| SSM_86_NDT_fwd | CCTGGAAGAATAACTACCGCAAC**NDT**CACTCCGCGACCACG |
| SSM_87_NDT_fwd | GGAAGAATAACTACCGCAACGCC**NDT**TCCGCGACCACGTGG |
| SSM_88_NDT_fwd | AGAATAACTACCGCAACGCCCAC**NDT**GCGACCACGTGGAGC |
| SSM_110_NDT_fwd | GGCGAGGATCAACACCCAGTGGCTG**NDT**ACCTTTGGCACCACCGAG |
| SSM_111_NDT_fwd | GAGGATCAACACCCAGTGGCTGCTG**NDT**TTTGGCACCACCGAGG |
| SSM_112_NDT_fwd | GATCAACACCCAGTGGCTGCTGACC**NDT**GGCACCACCGAGGC |
| SSM_113_NDT_fwd | CAACACCCAGTGGCTGCTGACCTTT**NDT**ACCACCGAGGCCAACG |
| SSM_114_NDT_fwd | CACCCAGTGGCTGCTGACCTTTGGC**NDT**ACCGAGGCCAACGCC |
| SSM_115_NDT_fwd | CCAGTGGCTGCTGACCTTTGGCACC**NDT**GAGGCCAACGCCTGG |
| SSM_117_NDT_fwd | GCTGCTGACCTTTGGCACCACCGAG**NDT**AACGCCTGGCAATCCAC |
| SSM_118_NDT_fwd | GCTGACCTTTGGCACCACCGAGGCC**NDT**GCCTGGCAATCCACGC |
| SSM_119_NDT_fwd | GACCTTTGGCACCACCGAGGCCAAC**NDT**TGGCAATCCACGCTGGT |
| SSM_120_NDT_fwd | CTTTGGCACCACCGAGGCCAACGCC**NDT**CAATCCACGCTGGTCG |
| SSM_121_NDT_fwd | TGGCACCACCGAGGCCAACGCCTGG**NDT**TCCACGCTGGTCGG |
| SSM_122_NDT_fwd | CACCACCGAGGCCAACGCCTGGCAA**NDT**ACGCTGGTCGGCCAC |
| SSM_123_NDT_fwd | CACCGAGGCCAACGCCTGGCAATCC**NDT**CTGGTCGGCCACGACAC |
| SSM_124_NDT_fwd | CGAGGCCAACGCCTGGCAATCCACG**NDT**GTCGGCCACGACACCTT |
| kanR_rev | CAACGGGAAACGTCTTGCTC |
| kanR_fwd | ATTTAATCGCGGCCTAGAGC |
| SSM_47_rev | GGCCGACTCGTAGGTTC |
| SSM_48_rev | GACGGCCGACTCGTAGG |
| SSM_49_rev | GCCGACGGCCGACTC |
| SSM_86_rev | GTTGCGGTAGTTATTCTTCCAGG |
| SSM_87_rev | GGCGTTGCGGTAGTTATTC |
| SSM_88_rev | GTGGGCGTTGCGGTAGTTAT |
| SSM_110_rev | CAGCCACTGGGTGTTGATC |
| SSM_111_rev | CAGCAGCCACTGGGTGTTG |
| SSM_112_rev | GGTCAGCAGCCACTGGG |
| SSM_113_rev | AAAGGTCAGCAGCCACTGG |
| SSM_114_rev | GCCAAAGGTCAGCAGCCAC |
| SSM_115_rev | GGTGCCAAAGGTCAGCAG |
| SSM_117_rev | CTCGGTGGTGCCAAAGGTC |

| Name | Sequence (5’ → 3’) |
|---|---|
| SSM_118_rev | GGCCTCGGTGGTGC |
| SSM_119_rev | GTTGGCCTCGGTGGTG |
| SSM_120_rev | GGCGTTGGCCTCGGTG |
| SSM_121_rev | CCAGGCGTTGGCCTCG |
| SSM_122_rev | TTGCCAGGCGTTGGCC |
| SSM_123_rev | GGATTGCCAGGCGTTGG |
| SSM_124_rev | CGTGGATTGCCAGGCGTTG |

**Table S3**. **Primers used to generate libraries of double, triple, quadruple and quintuple mutants.** Degenerate codons are highlighted in bold.

| Name | Sequence (5’ → 3’) |
|---|---|
| Sav_FQ_fwd | GGCACCACCGAGGCCAACGCCTGGCAATCCACGCTGGTCGGC |
| Sav_118X_fwd | GGCACCACCGAGGCC**NNK**GCCTGGCAATCCACGCTGGTCGGC |
| Sav_119X_fwd | GGCACCACCGAGGCCAAC**NNK**TGGCAATCCACGCTGGTCGGC |
| Sav_121X_fwd | GGCACCACCGAGGCCAACGCCTGG**NNK**TCCACGCTGGTCGGC |
| Sav_118X_119X_fwd | GGCACCACCGAGGCC**NNKNNK**TGGCAATCCACGCTGGTCGGC |
| Sav_118X_121X_fwd | GGCACCACCGAGGCC**NNK**GCCTGG**NNK**TCCACGCTGGTCGGC |
| Sav_119X_121X_fwd | GGCACCACCGAGGCCAAC**NNK**TGG**NNK**TCCACGCTGGTCGGC |
| Sav_118X_119X_121X_fwd | GGCACCACCGAGGCC**NNKNNK**TGG**NNK**TCCACGCTGGTCGGC |
| Sav_FQ_rev | GGCCTCGGTGGTGCCAAAGGTCAGCAGCCACTGGGTG |
| Sav_111X_rev | GGCCTCGGTGGTGCCAAA**MNN**CAGCAGCCACTGGGTG |
| Sav_112X_rev | GGCCTCGGTGGTGCC**MNN**GGTCAGCAGCCACTGGGTG |
| Sav_111X_112X_rev | GGCCTCGGTGGTGCC**MNNMNN**CAGCAGCCACTGGGTG |
| kanR_fwd | ATTTAATCGCGGCCTAGAGC |
| kanR_rev | CAACGGGAAACGTCTTGCTC |

**Table S4. Primer combinations used to generate fragments with mutations at positions 111 and 112 or positions 118, 119, and 121.** The primer sequences can be found in Table S3.

| | Forward primer(s) | Reverse primer(s) |
|---|---|---|
| PCR 1 | Sav_FQ_fwd | kanR_rev |
| PCR 2 | Sav_118X_fwd<br>Sav_119X_fwd<br>Sav_121X_fwd | kanR_rev |
| PCR 3 | Sav_118X_119X_fwd<br>Sav_118X_121X_fwd<br>Sav_119X_121X_fwd | kanR_rev |
| PCR 4 | Sav_118X_119X_121X_fwd | kanR_rev |
| PCR 5 | kanR_fwd | Sav_FQ_rev |
| PCR 6 | kanR_fwd | Sav_111X_rev<br>Sav_112X_rev |
| PCR 7 | kanR_fwd | Sav_111X_112X_rev |

**Table S5. Assembly scheme for fragments from Table S4.**

| Assembly reaction | Type of mutants | Fragment A | Fragment B | Theoretical diversity |
|---|---|---|---|---|
| 1 | Double | PCR 7 | PCR 1 | 400 |
| 2 | Double | PCR 6 | PCR 2 | 2,400 |
| 3 | Double | PCR 5 | PCR 3 | 1,200 |
| 4 | Triple | PCR 7 | PCR 2 | 24,000 |
| 5 | Triple | PCR 6 | PCR 3 | 48,000 |
| 6 | Triple | PCR 5 | PCR 4 | 8,000 |
| 7 | Quadruple | PCR 7 | PCR 3 | 480,000 |
| 8 | Quadruple | PCR 6 | PCR 4 | 320,000 |
| 9 | Quintuple | PCR 7 | PCR 4 | 3,200,000 |

**Table S6. Primers used to create libraries of specific variants suggested by the machine learning model.** The positions at which the oligo pools contained diverse nucleotides are represented by Ns and are highlighted in bold.

| Description | Sequence (5' → 3') |
|---|---|
| Oligo pool | GAGGATCAACACCCAGTGGCTGCTG**NNNNNN**GGCACCACCGAGGCC**NNNNNN**TG G**NNN**TCCACGCTGGTCGGCCACG |
| kanR_rev_B | CAACGGGAAACGTCTTGCTCTAGGCC |
| ML_amp_fwd | GAGGATCAACACCCAGTGGC |
| kanR_rev | CAACGGGAAACGTCTTGCTC |
| kanR_fwd | ATTTAATCGCGGCCTAGAGC |
| SSM_111_rev | CAGCAGCCACTGGGTGTTG |

**Table S7. PCRs conducted to clone the first library of ML-designed Sav variants.** For PCR 1, 15 cycles were performed with the initial primer combination, at which point the other primers were added for 20 additional cycles to amplify the product further. The primer sequences can be found in Table S6.

| | Forward primer | Concentration | Reverse primer | Concentration | Cycles |
|---|---|---|---|---|---|
| PCR 1 | Oligo pool | 2 nM | kanR_rev_B | 500 nM | 15 |
| | ML_amp_fwd | 500 nM | kanR_rev | 500 nM | 20 |
| PCR 2 | kanR_fwd | 500 nM | SSM_111_rev | 500 nM | 30 |

**Table S8. PCRs conducted to clone the second library of ML-designed Sav variants.** The primer sequences can be found in Table S6.

| | Forward primer | Concentration | Reverse primer | Concentration | Cycles |
|---|---|---|---|---|---|
| PCR 1 | Oligo pool | 40 nM | kanR_rev_B | 500 nM | 35 |
| PCR 2 | kanR_fwd | 500 nM | SSM_111_rev | 500 nM | 30 |

**Table S9. Primers for well-specific barcoding of Sav mutants.** Barcodes are highlighted in bold.

| Description | Sequence (5’ → 3’) |
|---|---|
| Sav_seq_rowA_fwd | GAGACCTTGTGTCGATGG**GGAGAA**GACCGGAACCTACGAGTCGGCC |
| Sav_seq_rowB_fwd | GAGACCTTGTGTCGATGG**CTGGAA**GACCGGAACCTACGAGTCGGCC |
| Sav_seq_rowC_fwd | GAGACCTTGTGTCGATGG**TCCGAA**GACCGGAACCTACGAGTCGGCC |
| Sav_seq_rowD_fwd | GAGACCTTGTGTCGATGG**ACAGTG**GACCGGAACCTACGAGTCGGCC |
| Sav_seq_rowE_fwd | GAGACCTTGTGTCGATGG**GTCTAG**GACCGGAACCTACGAGTCGGCC |
| Sav_seq_rowF_fwd | GAGACCTTGTGTCGATGG**CTCTTC**GACCGGAACCTACGAGTCGGCC |
| Sav_seq_rowG_fwd | GAGACCTTGTGTCGATGG**TATCGC**GACCGGAACCTACGAGTCGGCC |
| Sav_seq_rowH_fwd | GAGACCTTGTGTCGATGG**AGTAGG**GACCGGAACCTACGAGTCGGCC |
| Sav_seq_col1_rev | GTCACACGTAGCATGTGG**CATAGG**CCTTGGTGAAGGTGTCGTGGCC |
| Sav_seq_col2_rev | GTCACACGTAGCATGTGG**TCCGTT**CCTTGGTGAAGGTGTCGTGGCC |
| Sav_seq_col3_rev | GTCACACGTAGCATGTGG**CATGCA**CCTTGGTGAAGGTGTCGTGGCC |
| Sav_seq_col4_rev | GTCACACGTAGCATGTGG**TTGTGG**CCTTGGTGAAGGTGTCGTGGCC |
| Sav_seq_col5_rev | GTCACACGTAGCATGTGG**TTGCCT**CCTTGGTGAAGGTGTCGTGGCC |
| Sav_seq_col6_rev | GTCACACGTAGCATGTGG**TTGGTC**CCTTGGTGAAGGTGTCGTGGCC |
| Sav_seq_col7_rev | GTCACACGTAGCATGTGG**CTCTTG**CCTTGGTGAAGGTGTCGTGGCC |
| Sav_seq_col8_rev | GTCACACGTAGCATGTGG**GGACAA**CCTTGGTGAAGGTGTCGTGGCC |
| Sav_seq_col9_rev | GTCACACGTAGCATGTGG**GACTTC**CCTTGGTGAAGGTGTCGTGGCC |
| Sav_seq_col10_rev | GTCACACGTAGCATGTGG**CCAATC**CCTTGGTGAAGGTGTCGTGGCC |
| Sav_seq_col11_rev | GTCACACGTAGCATGTGG**CAACGA**CCTTGGTGAAGGTGTCGTGGCC |
| Sav_seq_col12_rev | GTCACACGTAGCATGTGG**AAGGCT**CCTTGGTGAAGGTGTCGTGGCC |

**Table S10. Primers for plate-specific barcoding of Sav mutants.** Barcodes are highlighted in bold.

| Description | Sequence (5’ → 3’) |
|---|---|
| Sav_seq_ext1_fwd | CAAGCAGAAGACGGCATACGAGATGTGACTGGAGTTCAGACGTGTGCTCTTCCGATCT**GGTAC**GAGACCTTGTGTCGATGG |
| Sav_seq_ext2_fwd | CAAGCAGAAGACGGCATACGAGATGTGACTGGAGTTCAGACGTGTGCTCTTCCGATCTC**AACAC**GAGACCTTGTGTCGATGG |
| Sav_seq_ext3_fwd | CAAGCAGAAGACGGCATACGAGATGTGACTGGAGTTCAGACGTGTGCTCTTCCGATCTAT**CGGTT**GAGACCTTGTGTCGATGG |
| Sav_seq_ext4_fwd | CAAGCAGAAGACGGCATACGAGATGTGACTGGAGTTCAGACGTGTGCTCTTCCGATCTTCG**GTCAA**GAGACCTTGTGTCGATGG |
| Sav_seq_ext5_fwd | CAAGCAGAAGACGGCATACGAGATGTGACTGGAGTTCAGACGTGTGCTCTTCCGATCTATCG**AAGCG**GAGACCTTGTGTCGATGG |
| Sav_seq_ext6_fwd | CAAGCAGAAGACGGCATACGAGATGTGACTGGAGTTCAGACGTGTGCTCTTCCGATCTG**CCACA**GAGACCTTGTGTCGATGG |
| Sav_seq_ext1_rev | AATGATACGGCGACCACCGAGATCTACACTCTTTCCCTACACGACGCTCTTCCGATCT**AGGAA**GTCACACGTAGCATGTGG |
| Sav_seq_ext2_rev | AATGATACGGCGACCACCGAGATCTACACTCTTTCCCTACACGACGCTCTTCCGATCTG**AGTGG**GTCACACGTAGCATGTGG |
| Sav_seq_ext3_rev | AATGATACGGCGACCACCGAGATCTACACTCTTTCCCTACACGACGCTCTTCCGATCTCC**ACGTC**GTCACACGTAGCATGTGG |
| Sav_seq_ext4_rev | AATGATACGGCGACCACCGAGATCTACACTCTTTCCCTACACGACGCTCTTCCGATCTTTC**TCAGC**GTCACACGTAGCATGTGG |
| Sav_seq_ext5_rev | AATGATACGGCGACCACCGAGATCTACACTCTTTCCCTACACGACGCTCTTCCGATCTCAAG**CTAGC**GTCACACGTAGCATGTGG |
| Sav_seq_ext6_rev | AATGATACGGCGACCACCGAGATCTACACTCTTTCCCTACACGACGCTCTTCCGATCTT**GCTTA**GTCACACGTAGCATGTGG |

**Table S11. Features used for the chemical descriptors.**

| Feature group name | Number of features | Description | Source |
|---|---|---|---|
| Z-scales | 8 | PCA score | [10] |
| VHSE | 5 | PCA score | [11] |
| Barley | 2 | PCA score | [12] |
| PCscores | 11 | PCA score | [13] |

**Table S12. Geometric features calculated for dimeric Sav.** Notice that these are raw features. Subsequently, we discarded features that did not vary across the 3.2 million structures, leaving us with 682 features. Solvent-accessible surface area is abbreviated SASA.

| Feature group name | Number of features | Description | Source |
|---|---|---|---|
| SASA 1.4 | 238 | Rolling ball alg. | Biotite[7] |
| SASA 1.4 H | 238 | Rolling ball alg. | Biotite |
| SASA 3.5 | 238 | Rolling ball alg. | Biotite |
| SASA 3.5 H | 238 | Rolling ball alg. | Biotite |
| SASA 5.5 | 238 | Rolling ball alg. | Biotite |
| SASA 5.5 H | 238 | Rolling ball alg. | Biotite |
| Partial charges | 238 | Gasteiger method | Biotite |
| Centre of mass | 238*3 | Min ball method | Minball |
| Radius | 238*3 | Min ball method | Minball |
| Dihedrals | 238 | | Biotite |
| Hydrogen bonds per residue | 238 | [23] | Biotite |

**Table S13. Ref2015 score components from the Rosetta Suite[24].** These scores were calculated for each approximate structure.

| Feature name | Description |
|---|---|
| fa_atr | Lennard-Jones attractive between atoms in different residues |
| fa_rep | Lennard-Jones repulsive between atoms in different residues |
| fa_sol | Lazaridis-Karplus solvation energy |
| fa_intra_sol_xover4 | Intra-residue Lazaridis-Karplus solvation energy |
| lk_ball_wtd | Asymmetric solvation energy |
| fa_intra_rep | Lennard-Jones repulsive between atoms in the same residue |
| fa_elec | Coulombic electrostatic potential with a distance-dependent dielectric |
| pro_close | Proline ring closure energy and energy of psi angle of preceding residue |
| hbond_sr_bb | Backbone-backbone hbonds close in primary sequence |
| hbond_lr_bb | Backbone-backbone hbonds distant in primary sequence |
| hbond_bb_sc | Sidechain-backbone hydrogen bond energy |
| hbond_sc | Sidechain-sidechain hydrogen bond energy |
| dslf_fa13 | Disulfide geometry potential |
| rama_prepro | Ramachandran preferences (with separate lookup tables for pre-proline positions and other positions) |
| omega | Omega dihedral in the backbone. A Harmonic constraint on planarity with standard deviation of ~6 deg. |
| p_aa_pp | Probability of amino acid, given torsion values for phi and psi |
| fa_dun | Internal energy of sidechain rotamers as derived from Dunbrack's statistics |
| yhh_planarity | A special torsional potential to keep the tyrosine hydroxyl in the plane of the aromatic ring |
| ref | Reference energy for each amino acid. Balances internal energy of amino acid terms. Plays a role in design. |

**Table S14. Allocation of the experimental budget in the exploration round.**

| Group | Number of Sav variants |
|---|---|
| Informative | 504 |
| Conservative prediction | 72 |
| Balanced prediction | 72 |
| Optimistic prediction | 72 |

**Table S15. Allocation of the experimental budget in the exploitation round.**

| Group | Model | Number of Sav variants |
|---|---|---|
| Conservative prediction | Chemical | 120 |
| Balanced prediction | Chemical | 120 |
| Conservative prediction | Geometric | 120 |
| Balanced prediction | Geometric | 120 |
| Conservative prediction | Energy-based | 120 |
| Balanced prediction | Energy-based | 120 |

# References


1. Vornholt, T. *et al.* Systematic engineering of artificial metalloenzymes for new-to-nature reactions. *Sci. Adv.* **7**, eabe4208 (2021).

2. Jeschek, M. *et al.* Directed evolution of artificial metalloenzymes for in vivo metathesis. *Nature* **537**, 661–665 (2016).

3. Piñero-Fernandez, S., Chimerel, C., Keyser, U. F. & Summers, D. K. Indole transport across Escherichia coli membranes. *J. Bacteriol.* **193**, 1793–1798 (2011).

4. Kada, G., Kaiser, K., Falk, H. & Gruber, H. J. Rapid estimation of avidin and streptavidin by fluorescence quenching or fluorescence polarization. *Biochim. Biophys. Acta* **1427**, 44–8 (1999).

5. Pedregosa, F. *et al.* Scikit-Learn: Machine learning in Python. *J. Mach. Learn. Res.* **12**, 2825–2830 (2011).

6. Paszke, A. *et al.* PyTorch: An imperative style, high-performance deep learning library. in *Advances in Neural Information Processing Systems 32* 8024–8035 (Curran Associates, Inc., 2019).

7. Kunzmann, P. & Hamacher, K. Biotite: a unifying open source computational biology framework in Python. *BMC Bioinformatics* **19**, 1–8 (2018).

8. Chaudhury, S., Lyskov, S. & Gray, J. J. PyRosetta: a script-based interface for implementing molecular modeling algorithms using Rosetta. *Bioinformatics* **26**, 689–691 (2010).

9. Virtanen, P. *et al.* SciPy 1.0: Fundamental Algorithms for Scientific Computing in Python. *Nat Methods* **17**, 261–272 (2020).

10. Sandberg, M., Eriksson, L., Jonsson, J., Sjöström, M. & Wold, S. New chemical descriptors relevant for the design of biologically active peptides. A multivariate characterization of 87 amino acids. *J. Med. Chem.* **41**, 2481–2491 (1998).

11. Mei, H., Liao, Z. H., Zhou, Y. & Li, S. Z. A new set of amino acid descriptors and its application in peptide QSARs. *Biopolymers* **80**, 775–786 (2005).

12. Barley, M. H., Turner, N. J. & Goodacre, R. Improved descriptors for the quantitative structure-activity relationship modeling of peptides and proteins. *J. Chem. Inf. Model.* **58**, 234–243 (2018).

13. Xu, Y. *et al.* Deep dive into machine learning models for protein engineering. *J. Chem. Inf. Model.* **60**, 2773–2790 (2020).

14. Lotfi, S., Izmailov, P., Benton, G., Goldblum, M. & Wilson, A. G. Bayesian model selection, the marginal likelihood, and generalization. in *Proceedings of the 39th International Conference on Machine Learning* (2022).

15. Rasmussen, C. E. & Williams, C. K. I. *Gaussian Processes for Machine Learning*. *The MIT Press, Cambridge, doi* (The MIT Press, 2005). doi:10.7551/mitpress/3206.001.0001.

16. Neal, R. M. *Bayesian Learning for Neural Networks*. vol. 118 (Springer Science & Business Media, 2012).

17. Srinivas, N., Krause, A., Kakade, S. M. & Seeger, M. W. Information-theoretic regret bounds for Gaussian process optimization in the bandit setting. *IEEE Trans. Inf. Theory* **58**, 3250–3265 (2012).

18. Kulesza, A. & Taskar, B. Determinantal point processes for machine learning. *Found. Trends Mach. Learn.* **5**, 123–286 (2012).

19. Nava, E., Mutný, M. & Krause, A. Diversified sampling for batched Bayesian optimization with determinantal point processes. *Proceedings of the 25th International Conference on Artificial Intelligence and Statistics (AISTATS)* (2022).

20. Nikolov, A. & Singh, M. Maximizing determinants under partition constraints. in *Proceedings of the forty-eighth annual ACM symposium on Theory of Computing* 192–201 (2016).

21. Kathuria, T., Deshpande, A. & Kohli, P. Batched Gaussian Process Bandit Optimization via Determinantal Point Processes. in *NIPS'16: Proceedings of the 30th International Conference on Neural Information Processing Systems* 4213–4221 (2016).

22. Van Der Maaten, L. & Hinton, G. Visualizing data using t-SNE. *J. Mach. Learn. Res.* **9**, 2579–2605 (2008).

23. Baker, E. N. & Hubbard, R. E. Hydrogen bonding in globular proteins. *Prog Biophys Mol Biol* **44**, 97–179 (1984).

24. Park, H. *et al.* Simultaneous optimization of biomolecular energy functions on features from small molecules and macromolecules. *J. Chem. Theory Comput.* **12**, 6201–6212 (2016).
